# Effects of a recent volcanic eruption on the isolated population of the iconic red-billed chough in La Palma, Canary Islands

**DOI:** 10.7717/peerj.18071

**Published:** 2024-09-30

**Authors:** Guillermo Blanco, Iñigo Palacios-Martínez, Óscar Frías, José L. González del Barrio, Martina Carrete

**Affiliations:** 1 Department of Evolutionary Ecology, Museo Nacional de Ciencias Naturales, Madrid, Madrid, Spain; 2 Department of Physical, Chemical and Natural Systems, Universidad Pablo de Olavide, Sevilla, Spain

**Keywords:** Breeding pairs, Communal roosts, La Palma, Canary Islands, Red-billed chough *Pyrrhocorax pyrrhocorax*, Tajogaite volcano

## Abstract

The effects of volcanic eruptions on ecosystems, particularly on oceanic islands, have been widely studied because of their important role in land formation, climate patterns and biological processes. Although these phenomena can lead to habitat loss, population decline and even species extinction, their effects on isolated populations, especially vertebrates, are not fully understood due to the paucity of pre-eruption data and *in situ* observations. Here, we assess the impact of the recent eruption of the Tajogaite volcano in La Palma, Canary Islands, on a unique population of red-billed choughs (*Pyrrhocorax pyrrhocorax*), an emblematic bird species that symbolises the natural heritage of the island. Pre- and post-eruption surveys showed that the eruption did not significantly affect the overall size or distribution of the population, although the number of choughs decreased after the eruption in the northern and central roosts, and increased in the southern ones. Although the eruption resulted in the release of toxic gases and ash, the observed changes in chough distribution and numbers could be attributed to environmental variability and the use of different foraging areas by individuals rather than direct effects of the volcano. The high mobility of choughs may have allowed them to avoid the negative effects of the volcano in the immediate aftermath of the eruption. Future studies are recommended to assess the long-term effects of volcanic ash on feeding habitats and the possible accumulation of contaminants, such as heavy metals, in the food chain. This will allow not only to monitor the presence of these compounds in ecosystems, but also to understand the response of this species to environmental changes and ensure its conservation.

## Introduction

Volcanism is a fundamental force in land formation, climate oscillations and biological processes such as evolution, speciation and biogeography (Sigurdsson et al., 2015). Oceanic islands of volcanic origin have made it possible to reconstruct the main events of biota colonization and succession and to evaluate the impact of eruptions on the ecology, population dynamics and conservation of isolated populations and endemic species (Whittaker & Fernández-Palacios, 2007). There are some documented cases of population declines or even local and total extinctions of species on oceanic islands due to habitat loss associated with volcanic eruptions (Mayr & Diamond, 2001; Hilton et al., 2003; Dalsgaard et al., 2007). In addition, the noxious gases, ash and pyroclastic material emitted can affect the immediate health status of directly exposed individuals (Crisafulli et al., 2015). In particular, gases and ashes can produce direct effects such as irritation and inflammation of the ocular and respiratory mucous membranes, affecting vision and respiration, which can cause serious health problems and even death (Crisafulli et al., 2015; Plaza et al., 2019). If ashes are deposited on nesting areas, they can cover the nests of species nesting in open fields or on trees, leading to breeding failure (Dalsgaard et al., 2007; Williams, Drummond & Buxton, 2010; Katrínardóttir et al., 2015). Persistent toxic or potentially harmful elements and minerals contained in these materials, like heavy metals, can affect living organisms in the short term, but also chronically through food chains, with notable effects on reproduction and survival (Katrínardóttir et al., 2015; Ermolin et al., 2018; Di Marzio et al., 2020). These effects have been documented by focusing on health conditions of a sample of individuals exposed to volcanic emissions or by evaluating the loss of suitable habitat depending on the geographical context and groups of organisms coinciding with contemporary eruptions (Elizalde, 2014; Crisafulli et al., 2015; Alarcón et al., 2016; Sánchez-Ramos & Navarro-Sigüenza, 2022). However, studies evaluating the impact of historical or recent volcanic eruptions on the population dynamics and long-term trends of species are scarce due to the lack of information on distribution, abundance, population characteristics and ecology of many island species under pre-eruption environmental conditions.

Over the past few centuries, the Canary Islands (Spain) have experienced several relatively well-documented volcanic eruptions, the most recent of which occurred on the westernmost and geologically youngest islands of the archipelago (Carracedo, 2008; Carracedo et al., 2022). The most recent eruptive event occurred between 19 September and 13 December 2021, and its main craters were located on the ridge of Cumbre Vieja on the island of La Palma (Carracedo et al., 2022). This volcano, called Tajogaite, had a major impact on a large area (1,241 ha) of the western slope of the island, causing severe damage to natural habitats, banana plantations and urban areas due to the lava flow (Rodríguez-Hernández et al., 2022). During the eruption, large quantities of toxic gases and ash were ejected into the atmosphere over long and variable distances, affecting large areas of La Palma (Córdoba-Jabonero et al., 2023; Shatto et al., 2024), and reaching other islands in the Canary archipelago, the Iberian Peninsula and even Central America, depending on the prevailing wind conditions and atmospheric currents (Carracedo et al., 2022; Milford et al., 2023; Salgueiro et al., 2023). The impact of these emissions on the vegetation and fauna in La Palma is currently being analysed by multidisciplinary teams, which will also make it possible to evaluate the colonisation process by the biota of the directly affected by the lava flow (Medina et al., 2023; Sangil et al., 2024). Studies published to date have focused on the impact caused by the lava flow (Medina, Acevedo & Nogales, 2021; Nogales et al., 2022), and by gases and ashes on the forests of the endemic Canary Island pine, *Pinus canariensis* (Weiser et al., 2022), and on fish (Caballero et al., 2023). However, no study has assessed the impact of the volcano on entire vertebrate populations beyond the areas directly occupied by lava.

**Figure 1:**
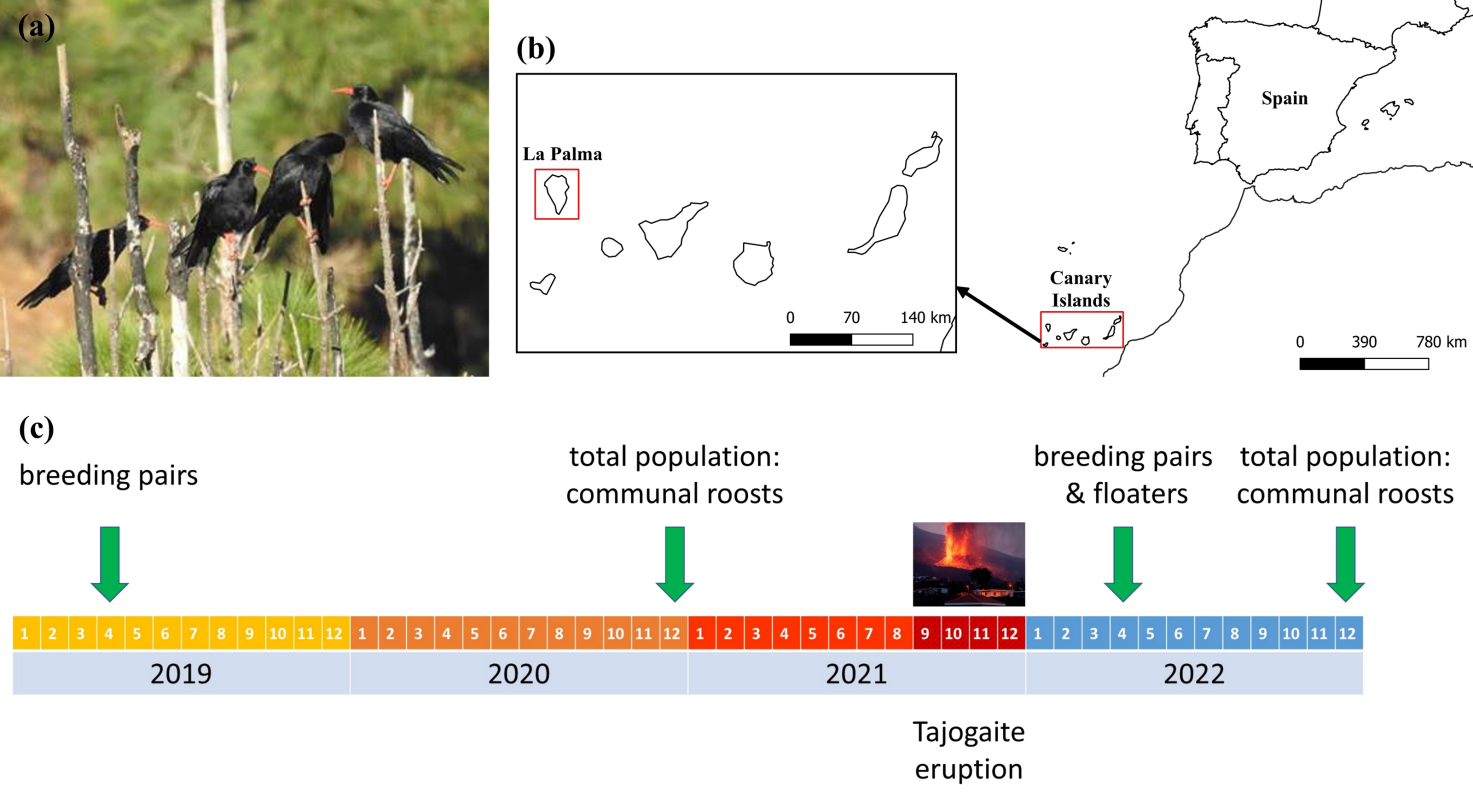
Localization of the study area, and chronological scheme of the censuses carried out. (A) Red-billed choughs perched on an endemic Canary Island pine, *Pinus canariensis*. (B) Study area showing the location of the island of La Palma within the Canary Archipelago. (C) Chronological scheme of the censuses carried out to evaluate the impact of the eruption of the Tajogaite volcano on the different population fractions. Estimates of the size of the floating population before the volcano were made in April 2004 (Blanco et al., 2009). Maps were elaborated with QGIS (Quantum Geographic Information System) v.3.22.7-Białowieża. (Image credit: Guillermo Blanco).

Among birds, the red-billed chough (*Pyrrhocorax pyrrhocorax,* Linnaeus, 1758; hereafter chough; Fig. 1A) is found only on the island of La Palma within the Canary archipelago, and its current population is isolated from the mainland (Morinha et al., 2020). Choughs are considered a symbol and “flagship” of the island’s natural values due to their beauty, charisma, and habits (Pais, 2005). Given that this species is distributed throughout the island and uses a wide variety of its habitats (Pais & García, 2000; Blanco, Laiolo & Fargallo, 2014), it can also be a good sentinel to assess the persistence of the effects of the volcano and its possible consequences on other species and geographical regions or ecosystems of the island. Furthermore, knowledge of the effects of the Tajogaite volcano on the distribution and abundance of this species is essential for assessing the current and future conservation of this isolated population.

In this study, we assessed the impact of the eruption of the Tajogaite volcano on the isolated and singular population of choughs on La Palma by using pre- and post-eruption surveys of the breeding, non-breeding and total populations. We tested the hypothesis that the eruption would have affected the population directly if the lava destroyed nesting, feeding or roosting areas. This hypothesis was also tested by considering the presumably more geographically widespread effects of ash distribution beyond the area affected by the lava flow. According to this hypothesis, the changes in the number of choughs using particular gorges as communal roosts and breeding sites before and after the eruption are predicted to be related to site characteristics, distance from the volcano and ash accumulation in their surroundings. Moreover, the volcano may have altered the current distribution of the species, and may have a potential influence on its future distribution and abundance, both in terms of the number of individuals concentrated in roosts and the number of breeding pairs remaining on the island after the eruption. Therefore, the effects of these same factors were considered in explaining the number of individuals remaining in each area of the island after the eruption.

## Materials & Methods

### Study area and species

The Canary Islands (Spain) form a volcanic archipelago belonging to the Macaronesian biogeographical region, consisting of seven main islands located in the Atlantic Ocean off the coast of northwest Africa. La Palma, located in the extreme northwest of the Canary archipelago (445 km from the African continent; Fig. 1B), is characterised by a high maximum altitude (Roque de Los Muchachos, 2,426 m above sea level) in a relatively small area (708 km^2^), which means that much of the island has steep slopes. This topography, together with the influence of the humidity brought by the prevailing north-east winds (trade winds), has created a wide variety of climatic conditions and ecosystems, characterised by a high level of biodiversity and a high proportion of endemic species of fauna and flora (Arechavaleta et al., 2010).

The chough is a highly social corvid with a fragmented distribution and several isolated populations. La Palma represents the south-western limit of its global distribution and is the only island currently inhabited by this species in the Canary archipelago (Fig. 1B), although its distribution was wider in the past, as evidenced by recent fossil records from La Gomera, Tenerife and El Hierro (Rando, 2007). Occasional observations have been reported in La Gomera and Tenerife, attributed to individuals moving temporarily from La Palma (58 km and 85 km from La Palma respectively), with no record of breeding establishment (Martín & Lorenzo, 2001). Both mitochondrial and nuclear data indicate that the chough population on La Palma is genetically well differentiated from those in Iberia and Morocco, and the best supported demographic model to explain the origin of La Palma choughs suggest the existence of a ‘ghost’ population closely related to Iberia, from which the insular population diverged within the last 30,000 years (Morinha et al., 2020). Therefore, La Palma population appears to be completely isolated, with no current contact with the nearest continental populations, which are located approximately 1,000 km away in the Atlas Mountains of Morocco (Morinha et al., 2020). The chough population on La Palma is one of the most important strongholds in the western Palaearctic and has the highest recorded population density (Blanco et al., 2007; Blanco et al., 2009). In recent decades, this population appears to have suffered a decline attributed to shooting, poisoning, habitat modification and the use of agricultural pesticides (Martín & Lorenzo, 2001; Pais, 2005; Blanco et al., 2007).

**Figure 2:**
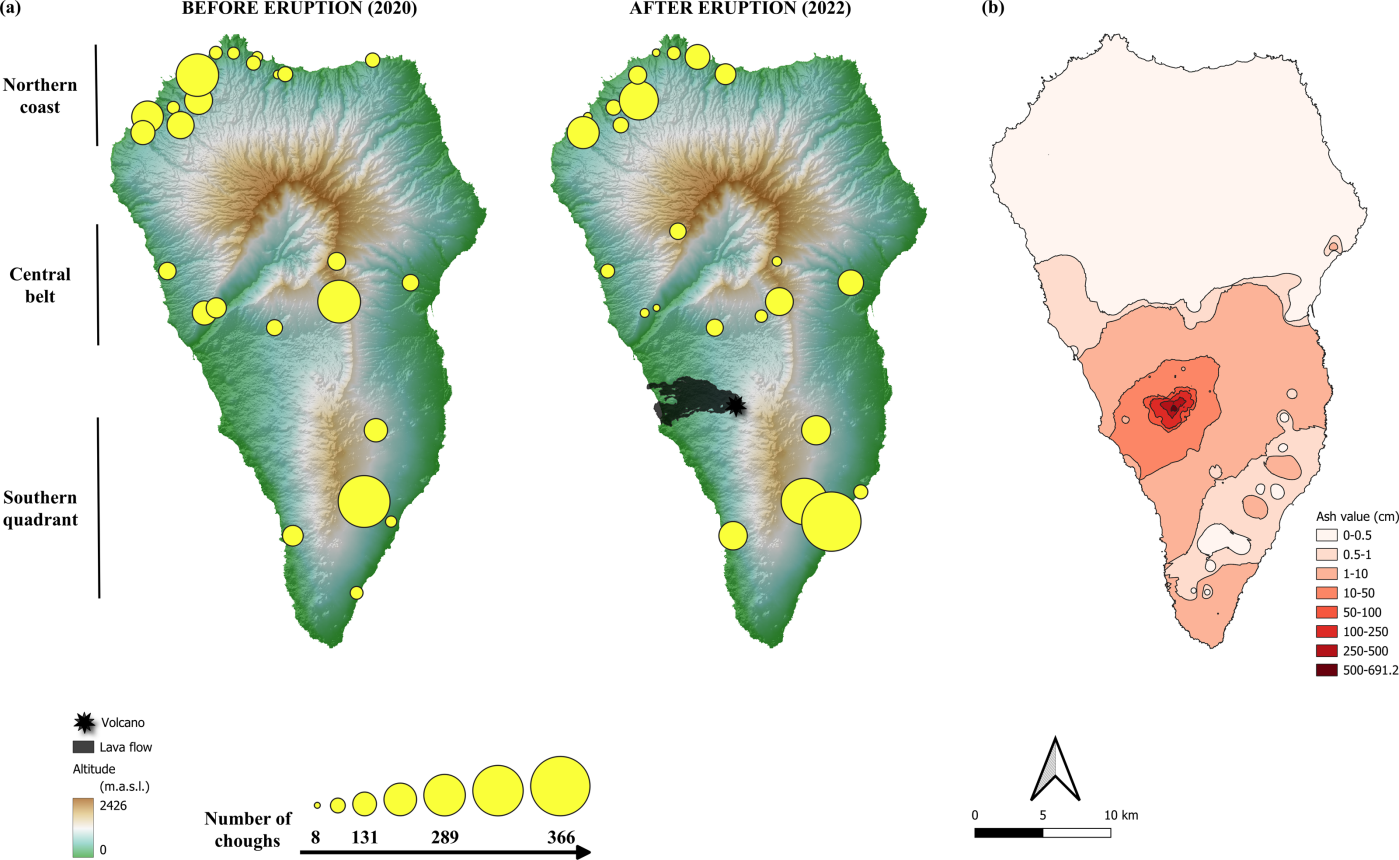
Location of red-billed chough communal roosts before and after the eruption of the Tajogaite volcano. (A) Location of red-billed chough communal roosts before and after the eruption of the Tajogaite volcano. The size of the circles is proportional to the number of individuals counted. The epicentre of the eruption and the lava flow are indicated. The background colours represent the altitude (in meters above sea level, m.a.s.l., Digital Surface Model MDS05, 5 m resolution; Centro Nacional de Información Geográfica, CNIG: http://centrodedescargas.cnig.es/). (B) Ash ejected by the volcano and accumulated along the island of La Palma according to data from Shatto et al. (2024). The colour scale indicates the interpolated values of tephra depth, ranging from shallow (light pink) to deep (dark red). Maps were elaborated with QGIS (Quantum Geographic Information System) v.3.22.7-Białowieża. (Image credit: Iñigo Palacios-Martínez).

Communal roosts of choughs congregate the entire non-breeding population throughout the year, and much of the breeding population during the non-breeding season. These sites represent important social points, with a role in the organization of diurnal foraging flocks and in mating (Blanco, Fargallo & Cuevas, 1993; Blanco & Tella, 1999). Previous studies have shown that communal roosts of choughs are distributed throughout the island, in both coastal and inland areas (Blanco et al., 2007; Blanco et al., 2009). The distribution of roosts suggest three main geographical population areas (Fig. 2A) associated with a geological division of the island: (1) the northern coast, consisting of roosts located mainly in coastal areas, distributed along the entire northern front and the northernmost quadrant of the west coast, the geologically oldest region of the island, (2) the central belt, consisting of a band that crosses the island from east to west, with inland roosts at either end located in ravines on the south-facing slopes of the Caldera de Taburiente, a huge depression in the centre of the island that structures its orography, and (3) the southern quadrant, which includes both coastal and inland roosts in the area of the island’s most recent volcanic activity (Carracedo, 2008).

### Censuses and quantification of population size

Choughs feed in flocks and form communal roosts composed of birds of different ages, including juveniles and sub-adults that make up the non-breeding floating population and a variable proportion of breeding pairs (Blanco & Tella, 1999; Blanco et al., 2009). By counting individuals roosting together at different times of the year, it is possible to estimate the size of the floating population during the breeding season and the total population in winter. Sampling to quantify the population of non-breeding individuals was carried out in April-early May (*i.e.,* in the core of the breeding season, lasting from March to June), as these individuals forage in flocks and always roost communally until they become breeders and acquire territory. During these months, breeding pairs incubate or care for nestlings, and failed breeding pairs continue to use their nesting sites for roosting (Blanco et al., 2009). Only a small proportion of breeding individuals that breed near roosts can be included in these counts (Blanco & Tella, 1999). Winter counts (December-January), on the other hand, are conducted when the number of birds at flocks is at its highest, as they gather all individuals in the population, both non-breeders and most breeders (Blanco, Fargallo & Cuevas, 1993).

Communal roosts may change location each year, so it is necessary to locate all roosts used each season by intensively monitoring of movements of flocks from foraging areas. To this end, coordinated censuses were carried out at all known sites used as communal roosts during the winter (total population) and breeding season (floating population) across the years. Surveys were conducted by a team of observers who simultaneously censused roosts in selected sectors of the island and monitor movements between nearby areas between days. One or two observers recorded the number of choughs at each roost as they returned (at dusk) or left (at dawn) the communal roosts. The number of individuals at each roost was recorded from places, ranging from 30 to 100 m to the roost cliff, which location changed in relation to changes in the areas selected by choughs for feeding at different times of the year. During the breeding season the start of activity and number of individuals in the roost were determined when there were more individuals than those from the pairs nesting in each roost site, generally coinciding with the arrival of flocks of variable size (Blanco, Fargallo & Cuevas, 1993; Blanco, Cuevas & Fargallo, 1993). Rainy, foggy or snowy days, generally during the non-breeding season, were avoided as they influence the arrival patterns of choughs at the roost, making it difficult to quantify flocks (Blanco, Cuevas & Fargallo, 1993).

A sample of cliffs used as communal roosts were monitored in April 2022 to quantify the number of breeding pairs, following standard procedures described by Blanco et al. (2009), and to compare with the number of pairs in those roosts before the volcano eruption (see next section). A territory was considered occupied by a breeding pair if individuals were observed building a nest, incubating, provisioning (*i.e.,* males providing food for females) or feeding nestlings. The observations to determine the number of breeding pairs were systematic, always using the same protocol until we were sure of the location of all of them on each sampled cliff. Although breeding continues in June, censuses of a sample of breeding pairs and the non-breeding population were carried out in April-early May depending on year, to try to avoid including in the census of the non-breeding population to breeding pairs that fail in their breeding attempts and then join the non-breeding flocks (Blanco, Fargallo & Cuevas, 1993).

### Population changes after the eruption

A chronological scheme of the censuses conducted to evaluate the impact of the eruption on the different population fractions is shown in Fig. 1C. To quantify the total population size, we conducted complete population surveys by counting communal roosts in December 2020 (pre-eruption winter season) and December 2022 (post-eruption winter season). Isolated pairs (up to a maximum of four) that used their nesting sites to roost on cliffs used also as communal roosts by flocks were not included in the analyses, because there are many other uncounted sites without communal roosts occupied by isolated pairs (Blanco et al., 2007; Blanco et al., 2009). The number of pairs roosting in isolation (outside communal roosts) in the non-breeding season was not quantified, as it is logistically challenging by means of a simultaneous island-wide census, and because this proportion may vary between years and even within a single non-breeding season (Blanco & Tella, 1999; Blanco, 2003). In a previous study, we quantified that about 12.5% of the pairs breeding outside communal roosts used their nesting sites as roosts during the non-breeding season (Blanco et al., 2009). In addition, communal roosting sites can change between years, but breeding pairs may keep their breeding places to roost in. In any case, this exclusion does not imply an important bias in the population quantification since the number of these isolated pairs represents a small proportion of the total wintering population (Blanco et al., 2009). The changes in the size and distribution of the non-breeding population mostly composed of floaters were assessed by comparing data from the complete census of communal roosts in the breeding season after the eruption (April-May 2022) with equivalent information obtained in 2004 (Blanco et al., 2009), 17 years before the eruption. Potential volcano-induced changes in the distribution and size of non-breeding roosts during the breeding season were not assessed due to the large time lag between the two monitoring periods. To assess changes in the breeding population, we compared the number of pairs nesting on the same monitored cliffs between April 2019 (penultimate pre-eruption breeding season) and April 2022 (post-eruption breeding season).

### Characteristics of communal roosts and ash accumulation

Using the Spanish Digital Surface Model (MDS05, 5 m resolution; Centro Nacional de Información Geográfica, CNIG: http://centrodedescargas.cnig.es/), we calculated the altitude and distance to the volcano (approximate centre of the main crater as a reference of the source of emissions) of the centre of each roost, as well as its orientation relative to the volcano (north, south, east, west, northeast, northeast, northeast, northwest, southeast, southwest), using the *raster* package (Hijmans, 2024). Similarly, we obtained the mean, minimum and maximum altitude of an imaginary line connecting each roost and the volcano as a measurement of the orographic barrier to the advance of emissions (orog mean, orog min and orog max, respectively). We assessed the potential impact of this measure as an estimate of emission exposure independent of the variability of the prevailing trade winds, the effect of which was assessed by taking into account the orientation of each roost with respect to the volcano (see above). We also estimated the amount of ash accumulated around the centre of each roost at 100 m, 500 m and 1,000 m radii using data from Shatto et al. (2024). The depth of the new ash layer across the island was quantified five months following the initial eruption, and it allows interpolation to the whole island territory (Shatto et al., 2024). All data were generated using QGIS software version 3.34.3 (https://www.qgis.org/).

### Statistical analysis

Linear models were used to test whether changes in the total number of individuals present at communal roosts and the number of breeding pairs after the eruption were related to the area in which the roosts or breeding pairs were located (northern coast, central belt or southern quadrant; fixed factor), the orientation of the roost location with respect to the volcano (fixed factor of eight categories: north, south, east, west, northeast, northeast, northeast, northwest, southeast, southwest), its altitude (continuous variable, in metres above sea level m. a.s.l.), the distance to the volcano (continuous variable, in metres m), the magnitude of orographic barriers (average, minimum or maximum altitude of an imaginary line connecting each roost and the volcano, in metres m) or the amount of ash accumulated in its surroundings (radii of 100 m, 500 m and 1,000 m; continuous variables). As the amount of ash accumulated at the different radii and the three variables describing the orography were correlated with each other, these variables were alternatively included in the models (*i.e.,* the models included one orography and ash variable at a time). Finally, as the number of individuals using each roost may vary for reasons unrelated to the volcanic eruption, we compared roost counts of the total population following the same methodology in winter 2004, 2009 and 2016 (Blanco et al., 2009) between the different areas (fixed factor) also using linear models (number of individuals).

Models were compared using the Akaike information criterion corrected for small sample sizes (AICc). Within each set of models (including the null model, but excluding models that did not converge), we calculated the ΔAICc as the difference between the AICc of model *i* and that of the best model and the Akaike weight (w) of each model. Models within 2 ΔAICc units of the best one were considered alternatives. We used this criterion because the relationships between our dependent and explanatory variables (and their combinations) are not well known, so we cannot have a restricted, pre-defined set of potential models. As model averaging has been questioned by several authors (*e.g.*, Cade, 2015; Banner & Higgs, 2017), we provide the output of the alternative models and discuss the importance of variables based on the significance (*p* < 0.05). All models were run using the glmmTMB package (Mollie et al., 2017) and their fit was evaluated using the DHARMa package (Hartig, 2022). Continuous variables were standardized before modelling and Tukey post hoc tests were used to compared fixed factor levels (lsmeans package; Lenth, 2016). All analyses were performed in R 4.0.1 (R Core Team, 2022).

## Results

The location of communal roosts and the number of choughs using them in the winter season before and one year after the eruption (December 2020 and 2022, respectively) are shown in Fig. 2A. The total number of roosts (pre-eruption: 24 roosts, post-eruption: 25 roosts) and their distribution in the three areas considered (northern coast, central belt, southern quadrant) did not differ between the two periods (Fisher’s Exact test: *p* = 0.597). No roosts or breeding colony were directly affected by the lava flow.

**Figure 3:**
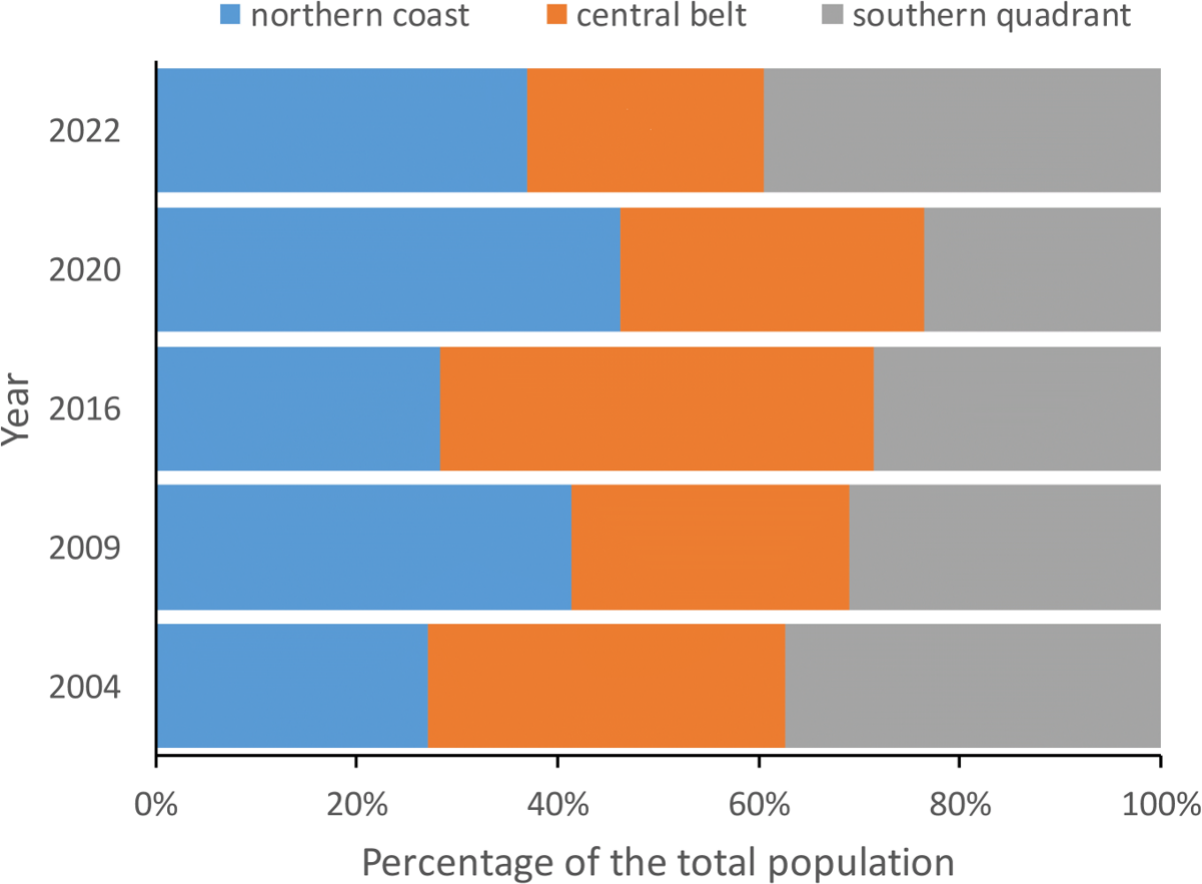
Percentage of red-billed choughs in communal roosts located in the northern coast, central belt and southern quadrant of the island of La Palma during the censuses carried out in winter 2004, 2009, 2016, 2020 and 2022 to estimate the total population size. The number of birds counted at communal roosts in each area (northern coast, central belt and southern quadrant) did not show significant differences in any of the years considered (2004: F_2,32_ =1.15, *p* = 0.3307, 2009: F_2,32_ =0.78, *p* = 0.4660, 2016: F_2,33_ =1.77, *p* = 0.1856, 2020: F_2,30_ =0.05, *p* = 0.9541 and 2022: F_2,33_ =2.16, *p* = 0.1318).

The total number of choughs present at winter communal roosts (total population size) was slightly higher before than after the eruption (2,667 and 2,546 individuals, respectively). Although the models show no significant differences between areas (Table 1; Fig. S1), the number of choughs decreased after the eruption in the northern and central roosts (−9.6% and −6.6%, respectively) and increased in the southern ones (+16.2%). However, such variation between areas is common, as shown by comparisons of the number of birds present at roosts between different years previous to the volcano eruption (Fig. 3). We found no significant effects of roost altitude or orientation, distance from the volcano, orographic barriers or nearby ash accumulation (Table 1; Fig. 2B). However, there is a non-significant trend for roosts closer to the volcano or located at higher altitudes to have more individuals during the winter after the eruption (Table 1; Fig. S2). The survey of all communal roosts (*n* =19) used during the post-volcanic breeding season (end of April 2022) revealed a total of 1,359 non-breeding floater individuals, representing 52.7% of the total population quantified in winter.

**Table 1:** Models showing relationships between explanatory variables on the change in the total number of choughs and the total number of choughs recorded after the eruption. Models obtained to assess the effect of the area where the communal roosts were located (northern coast, central belt or southern quadrant), altitude, orientation (north, south, east, west, northeast, northeast, northeast, northwest, southeast, southwest), orography (measured as maximum, mean and minimum altitude with respect to the volcano: orog max, orog mean and orog min, respectively; see Methods), distance from the volcano (distance) and amount of ash accumulated in the surroundings (100 m, 500 m and 1,000 m radii, ash 100 m, ash 500 m and ash 1,000 m, respectively) on the change in the total number of choughs and the total number of choughs recorded after the eruption.

Model	df	AICc	ΔAICc	w		Variables	Estimate	SE	F	*p*
Change in the number of total individuals										
null	2	339.07	0.00	0.10		area			2.03	0.1522
area	4	340.11	1.04	0.06						
distance	3	341.09	2.02	0.04						
ash 500m	3	341.32	2.25	0.03						
ash 100m	3	341.33	2.26	0.03						
orog min	3	341.34	2.27	0.03						
ash 1,000 m	3	341.37	2.30	0.03						
altitude	3	341.39	2.32	0.03						
orog mean	3	341.52	2.45	0.03						
orog max	3	341.52	2.45	0.03						
Number of total individuals after the eruption										
distance	3	114.55	0.00	0.07		distance	−0.55	0.32	−1.72	0.0850
altitude	3	114.71	0.17	0.07		altitude	0.53	0.32	1.67	0.0954
null	2	114.85	0.30	0.06		orog min	0.49	0.32	1.51	0.1320
orog min	3	115.18	0.63	0.05		orog max	−0.35	0.33	−1.05	0.2920
orog max	3	116.28	1.73	0.03		altitude	0.51	0.31	1.64	0.1020
altitude + orog max	4	116.46	1.91	0.03		orog max	−0.32	0.31	−1.01	0.3140
altitude + distance	4	116.57	2.03	0.03						
distance+ ash 100m	4	116.58	2.04	0.03						
ash 1,000 m	3	116.71	2.17	0.02						
orog min + distance	4	116.83	2.28	0.02						

**Notes.**

df number of estimated parameters AICc Akaike information criterion corrected for small sample sizes ΔAICc difference between each model and the best model (*i.e.* the model with the lowest AICc) weight Akaike’s weight. The significance of the variables (estimates, SE standard errors, statistic and p-value) are given only for the alternative models (ΔAICc <2) null null model. Only the first ten models are included, the rest can be found in Table S1

The number of breeding pairs at the monitored sites also decreased after the volcanic eruption (pre-eruption: 121 breeding pairs; post-eruption: 95 breeding pairs) following a similar pattern to the population as a whole. While the number of breeding pairs decreased in the north and centre (−40.74 and −24.14%, respectively), there was a slight increase in the south (7.89%). None of the variables considered were related to these changes (Table 2; Fig. S3), although the number of breeding pairs after the eruption was significantly higher in the south than in the other areas (Table 2; *a posteriori* comparisons between areas: central belt - northern coast: *p* = 0.6999, central belt - southern quadrant: *p* = 0.0005, northern coast - south: *p* = 0.0001; Fig. S4).

## Discussion

Volcanic eruptions can have direct effects on individuals, affecting their health, survival and reproduction, or indirect effects by altering the conditions or quality of the habitats they use to meet their needs at different stages of their life cycle (Sánchez-Ramos & Navarro-Sigüenza, 2022; Almaraz & Green, 2024). Determining the impact of volcanism on the persistence of isolated populations is therefore a challenge, often exacerbated by the general lack of historical data on many island populations, and the scarcity of *in situ* information during and after eruptive processes. We conducted a simultaneous counting on all communal roosts used during the winter by red-billed choughs on the oceanic island of La Palma after the Tajogaite eruption. This census represents an estimation of the total population size because during the winter the vast majority of individuals use communal roosts (Blanco et al., 2009). By comparing this census with previous ones using the same methodology, we show that the recent eruption of the Tajogaite volcano does not appear to have had a significant effect on the overall population size, although it may have contributed to the redistribution of individuals between different areas of the island. These results highlight the need to obtain information on the status and ecology of sensitive species in isolated populations on volcanically active islands in order to assess the impact of future eruptions.

**Table 2:** Models showing the relationships of explanatory factors on the number of breeding pairs. Models obtained to assess the effect of the area where the breeding pairs were located (northern coast, central belt or southern quadrant), altitude, orientation (north, south, east, west, northeast, northeast, northeast, northwest, southeast, southwest), orography (measured as maximum, mean and minimum altitude with respect to the volcano: orog max, orog mean and orog min, respectively; see Methods), distance from the volcano (distance) and amount of ash accumulated in the surroundings (100 m, 500 m and 1,000 m radii, ash 100 m, ash 500 m and ash 1,000 m, respectively) on the change in the total number of breeding pairs and the number of breeding pairs recorded after the eruption.

Model	df	AICc	ΔAICc	w		Variables	Estimate	SE	z	*p*
Change in the number of breeding pairs										
null	2	63.93	0.00	0.30		orog min	1.74	0.95	1.82	0.0682
orog min	3	64.95	1.02	0.18		distance	−1.54	0.99	−1.56	0.1182
distance	3	65.65	1.72	0.13		altitude	1.49	0.99	1.51	0.1319
altitude	3	65.79	1.86	0.12						
orog med	3	67.09	3.16	0.06						
ash 1,000 m	3	67.42	3.49	0.05						
ash 100 m	3	67.42	3.49	0.05						
ash 500 m	3	67.42	3.49	0.05						
orog max	3	67.79	3.87	0.04						
area	4	69.07	5.14	0.02						
Number of breeding pairs after the eruption										
area	4	59.66	0.00	1.00		area			36.6	<0.0001
orog min	3	72.62	12.96	0.00						
distance	3	74.53	14.87	0.00						
null	2	75.98	16.32	0.00						
orientation	6	76.25	16.58	0.00						
altitude	3	76.34	16.68	0.00						
ash 1,000 m	3	76.36	16.70	0.00						
ash 100 m	3	76.40	16.74	0.00						
ash 500 m	3	76.43	16.76	0.00						
orog med	3	78.75	19.09	0.00						

**Notes.**

df number of estimated parameters AICc Akaike information criterion corrected for small sample sizes ΔAICc difference between each model and the best model (*i.e.* the model with the lowest AICc) weight Akaike’s weight. The significance of the variables (estimates, SE standard errors, statistic and p-value) are given only for the alternative models ( ΔAICc <2) null null model. Only the first ten models are included, the rest can be found in Table S2.

The winter roosts of choughs on La Palma prior to the eruption of the Tajogaite volcano were located on cliffs and ravines in three main areas that were consistently occupied across the years (Blanco et al., 2007; Blanco et al., 2009). Neither roosts nor breeding colonies were directly affected by the lava flow. Although the lava destroyed banana plantations and urbanized areas generally not used as foraging grounds by choughs, a relatively small proportion of potential feeding areas on pine forests and scrubland were directly affected. In addition, the eruption distributed huge amounts of ash over a large part of the island, especially in the centre and southwest (Shatto et al., 2024), which requires specific research for a thorough assessment of the impact on chough foraging areas.

Overall, the total population size recorded in the winter censuses were slightly lower after than before the eruption. However, the amount of ash accumulated around roosts or other variables considered here (*e.g.*, ash accumulation, orientation, orography or distance from the volcano) were not associated with changes in either the difference between the number of individuals present in each roost before and after the eruption or the number of birds congregating at each roost after the eruption. Furthermore, the tendency for roosts with the highest numbers of individuals in the winter after the eruption to be those closest to the volcano suggests that even other effects, such as the release of toxic gases, might have been short-term and localized. Thus, the observed changes may be due to population fluctuations caused by other factors, such as inter-annual variability in environmental conditions and their effects on reproduction and survival (Reid et al., 2003; Banda & Blanco, 2009), or weather conditions at the time of sampling, which may affect the short-term distribution of individuals (Blanco et al., 2009). In fact, the relatively small size of La Palma and the great capacity for daily movement of choughs (Morinha et al., 2017a; Morinha et al., 2017b) allows them to travel over a large part of its surface to feed, which favours the frequent roost changes by some individuals (Blanco et al., 2007; Blanco et al., 2009). In addition, it would also allow to avoid volcano emissions depending on the changing wind direction. This aspect could also explain why we recorded more birds in the southern roosts located in the eastern slopes, since the prevailing winds favoured a greater accumulation of ash on the western slopes (Shatto et al., 2024), not only to the detriment of the central roosts (the most affected by ash, as assessed by its accumulation), but also those in the north (the least affected).

Monitoring of a sample of breeding colonies showed a higher number of breeding pairs before than after the eruption, but this was not related to the variables considered. However, while colonies in the central belt and northern coast showed a decreasing trend, those in the south increased after the eruption. Therefore, as in the case of communal roosts, the lower number of pairs after the eruption could be due to factors external to the volcano, such as seasonal environmental conditions or, more likely, habitat saturation, as suggested by the high proportion of non-breeding floaters present in this population (Blanco et al., 2009; Blanco, Laiolo & Fargallo, 2014; present data). In this regard, it should be noted that the pre-eruption census of breeding pairs corresponds to several years before the eruption, so comparisons with post-eruption counts and the influence of the eruption on the total population size (winter census) due to productivity should be made with caution. In any case, although there are fluctuations in the number of individuals, the location of roosts and breeding colonies was very stable over the years, suggesting a preference for certain sites that are used repeatedly, a common behaviour in the species (Blanco, Sánchez-Marco & Negro, 2021). Due to the life history strategy of this long-lived bird, the impact of productivity on total population size, as assessed by winter roost census, may be less than that of the eruptive process directly on adult survival. However, we lack information on breeding productivity data for these years, which prevent us from assessing these possibilities. Future studies should focus on determining whether there is a temporal component to the survival of choughs individually marked with distance-reading rings in previous years, and more accurately assess the impact of the Tajogaite volcano on the demography and population dynamics of this species.

## Conclusions

Our results suggest that the Tajogaite eruption does not appear to have had a significant impact on the distribution and abundance of breeding and non-breeding choughs, nor on population size, in La Palma. To our knowledge, this is the first study to assess the impact of this volcano on vertebrates at the population level. In this sense, our study species is particularly relevant because the study species is considered a symbol of the island and, due to its high mobility, can act as a bioindicator or sentinel of the long-term effects of the volcano beyond the area directly affected by the lava flow on other species. Future studies should focus on the long-term effects of accumulated ash on foraging habitats and the possible bottom-up transfer of compounds with potential health impacts, such as heavy metals, which accumulate in choughs and other species through food chains. Finally, it would also be essential to continue regular monitoring of this population, not only for its interest as a sentinel but also for its own conservation value.

##  Supplemental Information

10.7717/peerj.18071/supp-1 Supplemental Information 1 Raw data used for statistical analysis

10.7717/peerj.18071/supp-2 Supplemental Information 2 Details of statistical model outputs

## Additional Information and Declarations

### Data Availability

The following information was supplied regarding data availability: The raw data are available in the Supplemental File.
