# Supplementary material for: Effects of a recent volcanic eruption on the isolated population of the iconic red-billed chough in La Palma, Canary Islands
Source: PeerJ. 2024 Sep 30;12:e18071. doi: 10.7717/peerj.18071 (PMC11448657; doi:10.7717/peerj.18071)

SUPPLEMENTARY MATERIAL

**Effects of a recent volcanic eruption on the isolated population of an iconic island bird on La Palma, Canary Islands**

Guillermo Blanco, Íñigo Palacios, Óscar Frías, José L. González del Barrio, Martina Carrete

**Table S1.** Models obtained to assess the effect of the area where the communal roosts were located (northern coast, central belt or southern quadrant), altitude, orientation (north, south, east, west, northeast, northeast, northeast, northwest, southeast, southwest), orography (measured as maximum, mean and minimum altitude with respect to the volcano: orog max, orog mean and orog min, respectively; see Methods), distance from the volcano (distance) and amount of ash accumulated in the surroundings (100m, 500m and 1,000m radii, ash 100m, ash 500m and ash 1000m, respectively) on the change in the total number of choughs and the total number of choughs recorded after the eruption. df: number of estimated parameters, AICc: Akaike information criterion corrected for small sample sizes, ΔAICc: difference between each model and the best model (i.e. the model with the lowest AICc), weight: Akaike's weight.

| **Change in the number of total individuals** |  |  |  |  |
| --- | --- | --- | --- | --- |
| **Model** | **df** | **AICc** | **∆AICc** | **w** |
| null | 2 | 339.07 | 0.00 | 0.10 |
| area | 4 | 340.11 | 1.04 | 0.06 |
| distance | 3 | 341.09 | 2.02 | 0.04 |
| ash 500m | 3 | 341.32 | 2.25 | 0.03 |
| ash 100m | 3 | 341.33 | 2.26 | 0.03 |
| orog min | 3 | 341.34 | 2.27 | 0.03 |
| ash 1000m | 3 | 341.37 | 2.30 | 0.03 |
| altitude | 3 | 341.39 | 2.32 | 0.03 |
| orog mean | 3 | 341.52 | 2.45 | 0.03 |
| orog max | 3 | 341.52 | 2.45 | 0.03 |
| area + ash 100m | 5 | 342.03 | 2.96 | 0.02 |
| area + orog min | 5 | 342.18 | 3.11 | 0.02 |
| area + distance | 5 | 342.31 | 3.24 | 0.02 |
| area + ash 500m | 5 | 342.34 | 3.27 | 0.02 |
| area + ash 1000m | 5 | 342.35 | 3.28 | 0.02 |
| orog min + distance | 4 | 342.61 | 3.54 | 0.02 |
| altitude + distance | 4 | 342.73 | 3.66 | 0.02 |
| area + altitude | 5 | 342.79 | 3.72 | 0.02 |
| area + orog max | 5 | 342.85 | 3.78 | 0.01 |
| area + orog mean | 5 | 343.10 | 4.03 | 0.01 |
| orog max + distance | 4 | 343.11 | 4.04 | 0.01 |
| orog min + ash 500m | 4 | 343.35 | 4.28 | 0.01 |
| orog min + ash 1000m | 4 | 343.42 | 4.35 | 0.01 |
| orog min + ash 100m | 4 | 343.43 | 4.36 | 0.01 |
| altitude + orog max + distance | 5 | 343.53 | 4.47 | 0.01 |
| orog mean + distance | 4 | 343.53 | 4.47 | 0.01 |
| altitude + ash 500m | 4 | 343.65 | 4.59 | 0.01 |
| altitude + ash 100m | 4 | 343.70 | 4.63 | 0.01 |
| altitude + ash 1000m | 4 | 343.71 | 4.64 | 0.01 |
| distance + ash 500m | 4 | 343.83 | 4.76 | 0.01 |
| distance + ash 100m | 4 | 343.83 | 4.76 | 0.01 |
| distance + ash 1000m | 4 | 343.83 | 4.76 | 0.01 |
| orog max + ash 500m | 4 | 343.85 | 4.78 | 0.01 |
| orog max + ash 100m | 4 | 343.87 | 4.80 | 0.01 |
| orog max + ash 1000m | 4 | 343.93 | 4.86 | 0.01 |
| orog mean + ash 500m | 4 | 343.96 | 4.89 | 0.01 |
| orog mean + ash 100m | 4 | 343.97 | 4.90 | 0.01 |
| altitude + orog mean | 4 | 344.01 | 4.94 | 0.01 |
| orog mean + ash 1000m | 4 | 344.02 | 4.95 | 0.01 |
| altitude + orog min | 4 | 344.07 | 5.00 | 0.01 |
| altitude + orog max | 4 | 344.08 | 5.01 | 0.01 |
| altitude + orog mean + distance | 5 | 344.16 | 5.09 | 0.01 |
| area + orog min + ash 100m | 6 | 344.81 | 5.75 | 0.01 |
| area + altitude + orog max | 6 | 345.08 | 6.01 | 0.00 |
| area + distance + ash 100m | 6 | 345.09 | 6.02 | 0.00 |
| area + orog min + ash 500m | 6 | 345.09 | 6.02 | 0.00 |
| area + altitude + ash 100m | 6 | 345.12 | 6.05 | 0.00 |
| area + orog min + ash 1000m | 6 | 345.13 | 6.06 | 0.00 |
| area + orog max + ash 100m | 6 | 345.15 | 6.08 | 0.00 |
| area + orog min + distance | 6 | 345.25 | 6.18 | 0.00 |
| area + orog mean + ash 100m | 6 | 345.30 | 6.23 | 0.00 |
| area + orog max + distance | 6 | 345.32 | 6.25 | 0.00 |
| area + distance + ash 500m | 6 | 345.35 | 6.28 | 0.00 |
| area + distance + ash 1000m | 6 | 345.38 | 6.31 | 0.00 |
| altitude + orog min + distance | 5 | 345.40 | 6.33 | 0.00 |
| area + altitude + orog min | 6 | 345.41 | 6.34 | 0.00 |
| orog min + orientation | 8 | 345.42 | 6.36 | 0.00 |
| area + altitude + ash 500m | 6 | 345.43 | 6.37 | 0.00 |
| area + orog max + ash 500m | 6 | 345.43 | 6.37 | 0.00 |
| area + orog max + ash 1000m | 6 | 345.44 | 6.37 | 0.00 |
| area + altitude + ash 1000m | 6 | 345.47 | 6.40 | 0.00 |
| area + orog mean + distance | 6 | 345.50 | 6.43 | 0.00 |
| area + altitude + distance | 6 | 345.54 | 6.48 | 0.00 |
| orog min + distance + ash 500m | 5 | 345.55 | 6.48 | 0.00 |
| orog min + distance + ash 1000m | 5 | 345.57 | 6.50 | 0.00 |
| orog min + distance + ash 100m | 5 | 345.57 | 6.50 | 0.00 |
| area + orog mean + ash 500m | 6 | 345.60 | 6.53 | 0.00 |
| area + orog mean + ash 1000m | 6 | 345.61 | 6.54 | 0.00 |
| area + altitude + orog mean | 6 | 345.64 | 6.57 | 0.00 |
| altitude + distance + ash 100m | 5 | 345.70 | 6.63 | 0.00 |
| altitude + distance + ash 1000m | 5 | 345.70 | 6.63 | 0.00 |
| altitude + distance + ash 500m | 5 | 345.71 | 6.64 | 0.00 |
| orientation | 7 | 345.90 | 6.83 | 0.00 |
| orog max + distance + ash 1000m | 5 | 346.08 | 7.01 | 0.00 |
| orog max + distance + ash 100m | 5 | 346.10 | 7.03 | 0.00 |
| orog max + distance + ash 500m | 5 | 346.10 | 7.03 | 0.00 |
| altitude + orog min + ash 500m | 5 | 346.34 | 7.27 | 0.00 |
| altitude + orog min + ash 1000m | 5 | 346.41 | 7.34 | 0.00 |
| altitude + orog max + ash 500m | 5 | 346.41 | 7.34 | 0.00 |
| altitude + orog min + ash 100m | 5 | 346.42 | 7.35 | 0.00 |
| altitude + orog mean + ash 500m | 5 | 346.42 | 7.35 | 0.00 |
| altitude + orog max + ash 100m | 5 | 346.47 | 7.40 | 0.00 |
| altitude + orog mean + ash 100m | 5 | 346.48 | 7.41 | 0.00 |
| altitude + orog max + ash 1000m | 5 | 346.48 | 7.41 | 0.00 |
| altitude + orog mean + ash 1000m | 5 | 346.48 | 7.42 | 0.00 |
| orog mean + distance + ash 1000m | 5 | 346.50 | 7.43 | 0.00 |
| orog mean + distance + ash 100m | 5 | 346.52 | 7.45 | 0.00 |
| orog mean + distance + ash 500m | 5 | 346.52 | 7.45 | 0.00 |
| altitude + orog max + distance + ash 100m | 6 | 346.53 | 7.46 | 0.00 |
| altitude + orog max + distance + ash 1000m | 6 | 346.61 | 7.54 | 0.00 |
| altitude + orog max + distance + ash 500m | 6 | 346.63 | 7.56 | 0.00 |
| altitude + orog mean + distance + ash 100m | 6 | 347.07 | 8.00 | 0.00 |
| altitude + orog mean + distance + ash 1000m | 6 | 347.14 | 8.07 | 0.00 |
| altitude + orog mean + distance + ash 500m | 6 | 347.17 | 8.10 | 0.00 |
| area + altitude + orog max + ash 100m | 7 | 348.07 | 9.00 | 0.00 |
| area + altitude + orog max + ash 500m | 7 | 348.34 | 9.27 | 0.00 |
| area + altitude + orog max + ash 1000m | 7 | 348.38 | 9.31 | 0.00 |
| area + altitude + orog mean + ash 100m | 7 | 348.38 | 9.31 | 0.00 |
| area + orog min + distance + ash 100m | 7 | 348.39 | 9.32 | 0.00 |
| area + altitude + orog min + ash 100m | 7 | 348.41 | 9.34 | 0.00 |
| area + altitude + orog max + distance | 7 | 348.49 | 9.42 | 0.00 |
| area + orog max + distance + ash 100m | 7 | 348.51 | 9.44 | 0.00 |
| area + altitude + distance + ash 100m | 7 | 348.63 | 9.56 | 0.00 |
| area + orog mean + distance + ash 100m | 7 | 348.64 | 9.58 | 0.00 |
| area + orog min + distance + ash 500m | 7 | 348.65 | 9.58 | 0.00 |
| altitude + orog min + distance + ash 500m | 6 | 348.67 | 9.60 | 0.00 |
| altitude + orog min + distance + ash 1000m | 6 | 348.67 | 9.60 | 0.00 |
| altitude + orog min + distance + ash 100m | 6 | 348.68 | 9.61 | 0.00 |
| area + altitude + orog min + ash 500m | 7 | 348.68 | 9.61 | 0.00 |
| area + altitude + orog mean + ash 500m | 7 | 348.69 | 9.62 | 0.00 |
| area + orog min + distance + ash 1000m | 7 | 348.69 | 9.62 | 0.00 |
| area + altitude + orog min + ash 1000m | 7 | 348.71 | 9.64 | 0.00 |
| area + altitude + orog mean + ash 1000m | 7 | 348.72 | 9.65 | 0.00 |
| area + orog max + distance + ash 500m | 7 | 348.74 | 9.67 | 0.00 |
| area + orog max + distance + ash 1000m | 7 | 348.76 | 9.70 | 0.00 |
| area + altitude + orog mean + distance | 7 | 348.79 | 9.72 | 0.00 |
| area + altitude + orog min + distance | 7 | 348.80 | 9.73 | 0.00 |
| area + orog mean + distance + ash 500m | 7 | 348.89 | 9.82 | 0.00 |
| area + altitude + distance + ash 500m | 7 | 348.90 | 9.83 | 0.00 |
| area + orog mean + distance + ash 1000m | 7 | 348.92 | 9.85 | 0.00 |
| area + altitude + distance + ash 1000m | 7 | 348.94 | 9.87 | 0.00 |
| altitude + orientation | 8 | 348.98 | 9.91 | 0.00 |
| altitude + orog min + orientation | 9 | 349.09 | 10.02 | 0.00 |
| orog mean + orientation | 8 | 349.25 | 10.18 | 0.00 |
| orog min + orientation + ash 500m | 9 | 349.47 | 10.40 | 0.00 |
| orog min + orientation + ash 1000m | 9 | 349.54 | 10.47 | 0.00 |
| orog min + distance + orientation | 9 | 349.55 | 10.48 | 0.00 |
| distance + orientation | 8 | 349.58 | 10.51 | 0.00 |
| orientation + ash 100m | 8 | 349.60 | 10.53 | 0.00 |
| orientation + ash 1000m | 8 | 349.66 | 10.59 | 0.00 |
| orientation + ash 500m | 8 | 349.73 | 10.66 | 0.00 |
| orog min + orientation + ash 100m | 9 | 349.74 | 10.67 | 0.00 |
| orog max + orientation | 8 | 349.75 | 10.68 | 0.00 |
| area + altitude + orog max + distance + ash 100m | 8 | 352.04 | 12.97 | 0.00 |
| area + altitude + orog mean + distance + ash 100m | 8 | 352.28 | 13.21 | 0.00 |
| area + altitude + orog max + distance + ash 500m | 8 | 352.29 | 13.22 | 0.00 |
| area + altitude + orog max + distance + ash 1000m | 8 | 352.32 | 13.25 | 0.00 |
| area + altitude + orog min + distance + ash 100m | 8 | 352.36 | 13.29 | 0.00 |
| orog mean + distance + orientation | 9 | 352.45 | 13.38 | 0.00 |
| area + altitude + orog mean + distance + ash 500m | 8 | 352.55 | 13.48 | 0.00 |
| area + altitude + orog mean + distance + ash 1000m | 8 | 352.59 | 13.52 | 0.00 |
| area + altitude + orog min + distance + ash 500m | 8 | 352.61 | 13.54 | 0.00 |
| area + altitude + orog min + distance + ash 1000m | 8 | 352.64 | 13.57 | 0.00 |
| altitude + orog mean + orientation | 9 | 352.92 | 13.85 | 0.00 |
| orog mean + orientation + ash 100m | 9 | 352.96 | 13.89 | 0.00 |
| orog mean + orientation + ash 1000m | 9 | 353.04 | 13.97 | 0.00 |
| orog max + distance + orientation | 9 | 353.05 | 13.98 | 0.00 |
| orog mean + orientation + ash 500m | 9 | 353.19 | 14.12 | 0.00 |
| altitude + orog max + orientation | 9 | 353.20 | 14.13 | 0.00 |
| altitude + orientation + ash 100m | 9 | 353.29 | 14.22 | 0.00 |
| altitude + orientation + ash 1000m | 9 | 353.35 | 14.28 | 0.00 |
| altitude + orientation + ash 500m | 9 | 353.38 | 14.31 | 0.00 |
| altitude + distance + orientation | 9 | 353.39 | 14.32 | 0.00 |
| orog max + orientation + ash 100m | 9 | 353.40 | 14.33 | 0.00 |
| orog max + orientation + ash 1000m | 9 | 353.46 | 14.39 | 0.00 |
| altitude + orog min + orientation + ash 500m | 10 | 353.50 | 14.43 | 0.00 |
| altitude + orog min + orientation + ash 1000m | 10 | 353.59 | 14.52 | 0.00 |
| orog max + orientation + ash 500m | 9 | 353.65 | 14.58 | 0.00 |
| altitude + orog min + orientation + ash 100m | 10 | 353.84 | 14.77 | 0.00 |
| distance + orientation + ash 100m | 9 | 353.96 | 14.89 | 0.00 |
| distance + orientation + ash 1000m | 9 | 353.99 | 14.92 | 0.00 |
| distance + orientation + ash 500m | 9 | 354.00 | 14.93 | 0.00 |
| altitude + orog min + distance + orientation | 10 | 354.02 | 14.95 | 0.00 |
| orog min + distance + orientation + ash 500m | 10 | 354.37 | 15.30 | 0.00 |
| orog min + distance + orientation + ash 1000m | 10 | 354.42 | 15.35 | 0.00 |
| orog min + distance + orientation + ash 100m | 10 | 354.49 | 15.42 | 0.00 |
| orog mean + distance + orientation + ash 100m | 10 | 357.36 | 18.29 | 0.00 |
| altitude + orog mean + distance + orientation | 10 | 357.38 | 18.31 | 0.00 |
| orog mean + distance + orientation + ash 500m | 10 | 357.39 | 18.32 | 0.00 |
| orog mean + distance + orientation + ash 1000m | 10 | 357.39 | 18.32 | 0.00 |
| altitude + orog mean + orientation + ash 100m | 10 | 357.46 | 18.39 | 0.00 |
| altitude + orog mean + orientation + ash 1000m | 10 | 357.57 | 18.50 | 0.00 |
| altitude + orog max + orientation + ash 100m | 10 | 357.65 | 18.59 | 0.00 |
| altitude + orog mean + orientation + ash 500m | 10 | 357.66 | 18.59 | 0.00 |
| altitude + orog max + orientation + ash 1000m | 10 | 357.77 | 18.70 | 0.00 |
| orog max + distance + orientation + ash 100m | 10 | 357.84 | 18.77 | 0.00 |
| altitude + orog max + orientation + ash 500m | 10 | 357.88 | 18.81 | 0.00 |
| orog max + distance + orientation + ash 1000m | 10 | 357.91 | 18.84 | 0.00 |
| altitude + orog max + distance + orientation | 10 | 357.94 | 18.87 | 0.00 |
| altitude + distance + orientation + ash 100m | 10 | 357.96 | 18.89 | 0.00 |
| orog max + distance + orientation + ash 500m | 10 | 357.96 | 18.89 | 0.00 |
| altitude + distance + orientation + ash 1000m | 10 | 358.10 | 19.03 | 0.00 |
| altitude + distance + orientation + ash 500m | 10 | 358.20 | 19.13 | 0.00 |
| altitude + orog min + distance + orientation + ash 500m | 11 | 358.34 | 19.28 | 0.00 |
| altitude + orog min + distance + orientation + ash 1000m | 11 | 358.50 | 19.43 | 0.00 |
| altitude + orog min + distance + orientation + ash 100m | 11 | 359.27 | 20.20 | 0.00 |
| altitude + orog mean + distance + orientation + ash 500m | 11 | 362.91 | 23.84 | 0.00 |
| altitude + orog mean + distance + orientation + ash 100m | 11 | 362.92 | 23.85 | 0.00 |
| altitude + orog mean + distance + orientation + ash 1000m | 11 | 362.94 | 23.87 | 0.00 |
| altitude + orog max + distance + orientation + ash 100m | 11 | 363.21 | 24.14 | 0.00 |
| altitude + orog max + distance + orientation + ash 1000m | 11 | 363.32 | 24.25 | 0.00 |
| altitude + orog max + distance + orientation + ash 500m | 11 | 363.41 | 24.34 | 0.00 |
| **Number of total individuals after the eruption** | | | | |
| **Model** | **df** | **AICc** | **∆AICc** | **w** |
| distance | 3 | 114.55 | 0.00 | 0.07 |
| altitude | 3 | 114.71 | 0.17 | 0.07 |
| null | 2 | 114.85 | 0.30 | 0.06 |
| orog min | 3 | 115.18 | 0.63 | 0.05 |
| orog max | 3 | 116.28 | 1.73 | 0.03 |
| altitude + orog max | 4 | 116.46 | 1.91 | 0.03 |
| altitude + distance | 4 | 116.57 | 2.03 | 0.03 |
| distance + ash 100m | 4 | 116.58 | 2.04 | 0.03 |
| ash 1000m | 3 | 116.71 | 2.17 | 0.02 |
| orog min + distance | 4 | 116.83 | 2.28 | 0.02 |
| ash 500m | 3 | 116.87 | 2.32 | 0.02 |
| distance + ash 500m | 4 | 116.96 | 2.41 | 0.02 |
| orog mean + distance | 4 | 117.02 | 2.48 | 0.02 |
| distance + ash 1000m | 4 | 117.09 | 2.55 | 0.02 |
| ash 100m | 3 | 117.12 | 2.57 | 0.02 |
| altitude + orog mean | 4 | 117.28 | 2.73 | 0.02 |
| orog max + distance | 4 | 117.28 | 2.73 | 0.02 |
| altitude + orog min | 4 | 117.31 | 2.76 | 0.02 |
| altitude + ash 1000m | 4 | 117.35 | 2.81 | 0.02 |
| orog mean | 3 | 117.35 | 2.81 | 0.02 |
| altitude + ash 500m | 4 | 117.39 | 2.85 | 0.02 |
| altitude + ash 100m | 4 | 117.45 | 2.90 | 0.02 |
| orog min + ash 100m | 4 | 117.90 | 3.36 | 0.01 |
| orog min + ash 1000m | 4 | 117.90 | 3.36 | 0.01 |
| orog min + ash 500m | 4 | 117.92 | 3.37 | 0.01 |
| area + distance | 5 | 117.92 | 3.37 | 0.01 |
| area + distance + ash 100m | 6 | 118.22 | 3.67 | 0.01 |
| area | 4 | 118.46 | 3.91 | 0.01 |
| orog max + ash 1000m | 4 | 118.75 | 4.21 | 0.01 |
| orog max + ash 500m | 4 | 118.86 | 4.31 | 0.01 |
| orog max + ash 100m | 4 | 118.98 | 4.44 | 0.01 |
| area + distance + ash 500m | 6 | 119.00 | 4.46 | 0.01 |
| orog min + distance + ash 100m | 5 | 119.00 | 4.46 | 0.01 |
| altitude + distance + ash 100m | 5 | 119.07 | 4.53 | 0.01 |
| orog mean + distance + ash 100m | 5 | 119.09 | 4.54 | 0.01 |
| area + distance + ash 1000m | 6 | 119.31 | 4.77 | 0.01 |
| area + altitude + orog max | 6 | 119.32 | 4.77 | 0.01 |
| altitude + orog max + distance | 5 | 119.32 | 4.77 | 0.01 |
| altitude + distance + ash 500m | 5 | 119.35 | 4.80 | 0.01 |
| area + altitude | 5 | 119.36 | 4.82 | 0.01 |
| orog min + distance + ash 500m | 5 | 119.37 | 4.83 | 0.01 |
| altitude + orog max + ash 100m | 5 | 119.39 | 4.84 | 0.01 |
| altitude + distance + ash 1000m | 5 | 119.43 | 4.89 | 0.01 |
| altitude + orog max + ash 500m | 5 | 119.44 | 4.89 | 0.01 |
| altitude + orog max + ash 1000m | 5 | 119.44 | 4.90 | 0.01 |
| orog mean + ash 1000m | 4 | 119.45 | 4.90 | 0.01 |
| orog min + distance + ash 1000m | 5 | 119.50 | 4.96 | 0.01 |
| orog mean + distance + ash 500m | 5 | 119.53 | 4.98 | 0.01 |
| altitude + orog min + distance | 5 | 119.53 | 4.99 | 0.01 |
| altitude + orog mean + distance | 5 | 119.55 | 5.00 | 0.01 |
| orog max + distance + ash 100m | 5 | 119.57 | 5.02 | 0.01 |
| orog mean + ash 500m | 4 | 119.61 | 5.06 | 0.01 |
| orog mean + distance + ash 1000m | 5 | 119.69 | 5.14 | 0.01 |
| orog mean + ash 100m | 4 | 119.85 | 5.31 | 0.00 |
| orog max + distance + ash 500m | 5 | 119.94 | 5.40 | 0.00 |
| orog max + distance + ash 1000m | 5 | 120.08 | 5.53 | 0.00 |
| area + orog min | 5 | 120.20 | 5.65 | 0.00 |
| altitude + orog mean + ash 1000m | 5 | 120.20 | 5.66 | 0.00 |
| altitude + orog mean + ash 500m | 5 | 120.23 | 5.69 | 0.00 |
| altitude + orog min + ash 1000m | 5 | 120.26 | 5.72 | 0.00 |
| altitude + orog mean + ash 100m | 5 | 120.27 | 5.72 | 0.00 |
| altitude + orog min + ash 500m | 5 | 120.28 | 5.74 | 0.00 |
| altitude + orog min + ash 100m | 5 | 120.29 | 5.75 | 0.00 |
| area + altitude + distance | 6 | 120.64 | 6.09 | 0.00 |
| area + orog max + distance | 6 | 120.75 | 6.20 | 0.00 |
| area + orog max + distance + ash 100m | 7 | 120.93 | 6.39 | 0.00 |
| area + orog max | 5 | 121.08 | 6.53 | 0.00 |
| area + orog mean + distance | 6 | 121.15 | 6.61 | 0.00 |
| area + orog min + distance | 6 | 121.16 | 6.62 | 0.00 |
| area + ash 100m | 5 | 121.18 | 6.63 | 0.00 |
| area + altitude + orog max + ash 100m | 7 | 121.35 | 6.80 | 0.00 |
| area + altitude + orog mean | 6 | 121.36 | 6.81 | 0.00 |
| area + orog mean | 5 | 121.36 | 6.82 | 0.00 |
| area + ash 500m | 5 | 121.41 | 6.87 | 0.00 |
| area + altitude + distance + ash 100m | 7 | 121.43 | 6.89 | 0.00 |
| area + ash 1000m | 5 | 121.45 | 6.90 | 0.00 |
| area + orog mean + distance + ash 100m | 7 | 121.67 | 7.13 | 0.00 |
| area + orog min + distance + ash 100m | 7 | 121.75 | 7.21 | 0.00 |
| area + orog max + distance + ash 500m | 7 | 121.81 | 7.26 | 0.00 |
| area + altitude + orog max + ash 500m | 7 | 121.99 | 7.44 | 0.00 |
| area + altitude + orog max + distance | 7 | 122.05 | 7.50 | 0.00 |
| area + altitude + ash 100m | 6 | 122.08 | 7.54 | 0.00 |
| area + altitude + distance + ash 500m | 7 | 122.17 | 7.63 | 0.00 |
| area + orog max + distance + ash 1000m | 7 | 122.19 | 7.64 | 0.00 |
| altitude + orog mean + distance + ash 100m | 6 | 122.21 | 7.66 | 0.00 |
| altitude + orog min + distance + ash 100m | 6 | 122.21 | 7.66 | 0.00 |
| area + altitude + orog max + ash 1000m | 7 | 122.21 | 7.66 | 0.00 |
| altitude + orog max + distance + ash 100m | 6 | 122.23 | 7.69 | 0.00 |
| area + altitude + orog max + distance + ash 100m | 8 | 122.41 | 7.87 | 0.00 |
| area + altitude + distance + ash 1000m | 7 | 122.43 | 7.89 | 0.00 |
| area + altitude + ash 500m | 6 | 122.44 | 7.89 | 0.00 |
| altitude + orog max + distance + ash 500m | 6 | 122.46 | 7.91 | 0.00 |
| area + orog mean + distance + ash 500m | 7 | 122.48 | 7.94 | 0.00 |
| altitude + orog max + distance + ash 1000m | 6 | 122.52 | 7.97 | 0.00 |
| altitude + orog min + distance + ash 500m | 6 | 122.52 | 7.98 | 0.00 |
| area + altitude + ash 1000m | 6 | 122.53 | 7.99 | 0.00 |
| area + orog min + distance + ash 500m | 7 | 122.53 | 7.99 | 0.00 |
| altitude + orog mean + distance + ash 500m | 6 | 122.54 | 8.00 | 0.00 |
| area + altitude + orog min | 6 | 122.62 | 8.07 | 0.00 |
| altitude + orog min + distance + ash 1000m | 6 | 122.62 | 8.08 | 0.00 |
| altitude + orog mean + distance + ash 1000m | 6 | 122.65 | 8.10 | 0.00 |
| area + orog min + ash 100m | 6 | 122.65 | 8.10 | 0.00 |
| area + orog mean + distance + ash 1000m | 7 | 122.81 | 8.27 | 0.00 |
| area + orog min + distance + ash 1000m | 7 | 122.83 | 8.29 | 0.00 |
| area + orog min + ash 500m | 6 | 123.11 | 8.57 | 0.00 |
| area + altitude + orog mean + distance | 7 | 123.16 | 8.62 | 0.00 |
| area + orog min + ash 1000m | 6 | 123.26 | 8.72 | 0.00 |
| area + altitude + orog max + distance + ash 500m | 8 | 123.28 | 8.74 | 0.00 |
| area + altitude + orog max + distance + ash 1000m | 8 | 123.59 | 9.04 | 0.00 |
| area + orog max + ash 100m | 6 | 124.00 | 9.45 | 0.00 |
| area + altitude + orog mean + distance + ash 100m | 8 | 124.03 | 9.48 | 0.00 |
| area + altitude + orog min + distance | 7 | 124.07 | 9.53 | 0.00 |
| area + altitude + orog mean + ash 100m | 7 | 124.22 | 9.67 | 0.00 |
| area + orog max + ash 500m | 6 | 124.29 | 9.74 | 0.00 |
| area + orog max + ash 1000m | 6 | 124.34 | 9.80 | 0.00 |
| area + orog mean + ash 100m | 6 | 124.35 | 9.81 | 0.00 |
| area + orog mean + ash 500m | 6 | 124.59 | 10.05 | 0.00 |
| area + orog mean + ash 1000m | 6 | 124.63 | 10.09 | 0.00 |
| area + altitude + orog mean + ash 500m | 7 | 124.64 | 10.09 | 0.00 |
| area + altitude + orog mean + ash 1000m | 7 | 124.76 | 10.22 | 0.00 |
| area + altitude + orog mean + distance + ash 500m | 8 | 124.82 | 10.28 | 0.00 |
| orientation | 7 | 125.03 | 10.48 | 0.00 |
| area + altitude + orog mean + distance + ash 1000m | 8 | 125.09 | 10.55 | 0.00 |
| area + altitude + orog min + distance + ash 100m | 8 | 125.38 | 10.83 | 0.00 |
| area + altitude + orog min + ash 100m | 7 | 125.55 | 11.00 | 0.00 |
| area + altitude + orog min + ash 500m | 7 | 125.95 | 11.40 | 0.00 |
| area + altitude + orog min + ash 1000m | 7 | 126.06 | 11.52 | 0.00 |
| area + altitude + orog min + distance + ash 500m | 8 | 126.11 | 11.57 | 0.00 |
| area + altitude + orog min + distance + ash 1000m | 8 | 126.37 | 11.82 | 0.00 |
| altitude + orientation | 8 | 126.49 | 11.95 | 0.00 |
| orog max + orientation | 8 | 126.85 | 12.30 | 0.00 |
| orog min + orientation | 8 | 127.57 | 13.03 | 0.00 |
| distance + orientation | 8 | 127.77 | 13.22 | 0.00 |
| orog mean + orientation | 8 | 128.56 | 14.02 | 0.00 |
| orientation + ash 1000m | 8 | 128.61 | 14.06 | 0.00 |
| orientation + ash 500m | 8 | 128.74 | 14.19 | 0.00 |
| altitude + orog max + orientation | 9 | 128.96 | 14.42 | 0.00 |
| orientation + ash 100m | 8 | 128.98 | 14.43 | 0.00 |
| altitude + orog mean + orientation | 9 | 130.10 | 15.55 | 0.00 |
| altitude + orog max + distance + orientation | 10 | 130.66 | 16.11 | 0.00 |
| orog max + orientation + ash 100m | 9 | 130.69 | 16.14 | 0.00 |
| altitude + orientation + ash 1000m | 9 | 130.86 | 16.31 | 0.00 |
| altitude + orientation + ash 100m | 9 | 130.88 | 16.34 | 0.00 |
| altitude + orientation + ash 500m | 9 | 130.89 | 16.35 | 0.00 |
| altitude + distance + orientation | 9 | 130.90 | 16.36 | 0.00 |
| altitude + orog min + orientation | 9 | 130.91 | 16.36 | 0.00 |
| orog max + orientation + ash 500m | 9 | 131.13 | 16.59 | 0.00 |
| orog max + orientation + ash 1000m | 9 | 131.20 | 16.65 | 0.00 |
| orog max + distance + orientation | 9 | 131.23 | 16.68 | 0.00 |
| distance + orientation + ash 100m | 9 | 131.51 | 16.96 | 0.00 |
| orog min + distance + orientation | 9 | 131.61 | 17.06 | 0.00 |
| orog min + orientation + ash 100m | 9 | 131.89 | 17.34 | 0.00 |
| orog min + orientation + ash 1000m | 9 | 131.98 | 17.44 | 0.00 |
| orog min + orientation + ash 500m | 9 | 131.99 | 17.45 | 0.00 |
| distance + orientation + ash 500m | 9 | 132.03 | 17.49 | 0.00 |
| distance + orientation + ash 1000m | 9 | 132.12 | 17.57 | 0.00 |
| orog mean + distance + orientation | 9 | 132.17 | 17.63 | 0.00 |
| altitude + orog max + orientation + ash 100m | 10 | 132.58 | 18.04 | 0.00 |
| orog mean + orientation + ash 1000m | 9 | 132.80 | 18.25 | 0.00 |
| orog mean + orientation + ash 500m | 9 | 132.88 | 18.34 | 0.00 |
| orog mean + orientation + ash 100m | 9 | 132.98 | 18.44 | 0.00 |
| altitude + orog max + orientation + ash 500m | 10 | 133.28 | 18.73 | 0.00 |
| altitude + orog max + orientation + ash 1000m | 10 | 133.39 | 18.84 | 0.00 |
| altitude + orog mean + distance + orientation | 10 | 134.14 | 19.59 | 0.00 |
| altitude + orog mean + orientation + ash 100m | 10 | 134.73 | 20.19 | 0.00 |
| altitude + orog mean + orientation + ash 500m | 10 | 135.00 | 20.45 | 0.00 |
| altitude + orog mean + orientation + ash 1000m | 10 | 135.02 | 20.48 | 0.00 |
| orog max + distance + orientation + ash 100m | 10 | 135.12 | 20.58 | 0.00 |
| altitude + distance + orientation + ash 100m | 10 | 135.70 | 21.15 | 0.00 |
| orog min + distance + orientation + ash 100m | 10 | 135.71 | 21.16 | 0.00 |
| altitude + distance + orientation + ash 1000m | 10 | 135.79 | 21.24 | 0.00 |
| altitude + orog min + orientation + ash 1000m | 10 | 135.80 | 21.25 | 0.00 |
| altitude + orog min + orientation + ash 100m | 10 | 135.80 | 21.26 | 0.00 |
| orog max + distance + orientation + ash 500m | 10 | 135.83 | 21.28 | 0.00 |
| altitude + orog min + orientation + ash 500m | 10 | 135.83 | 21.29 | 0.00 |
| altitude + distance + orientation + ash 500m | 10 | 135.83 | 21.29 | 0.00 |
| altitude + orog min + distance + orientation | 10 | 135.84 | 21.29 | 0.00 |
| altitude + orog max + distance + orientation + ash 1000m | 11 | 135.94 | 21.39 | 0.00 |
| orog max + distance + orientation + ash 1000m | 10 | 135.95 | 21.40 | 0.00 |
| altitude + orog max + distance + orientation + ash 500m | 11 | 136.05 | 21.51 | 0.00 |
| altitude + orog max + distance + orientation + ash 100m | 11 | 136.21 | 21.67 | 0.00 |
| orog min + distance + orientation + ash 500m | 10 | 136.28 | 21.73 | 0.00 |
| orog min + distance + orientation + ash 1000m | 10 | 136.38 | 21.84 | 0.00 |
| orog mean + distance + orientation + ash 100m | 10 | 136.44 | 21.89 | 0.00 |
| orog mean + distance + orientation + ash 500m | 10 | 136.96 | 22.42 | 0.00 |
| orog mean + distance + orientation + ash 1000m | 10 | 137.04 | 22.50 | 0.00 |
| altitude + orog mean + distance + orientation + ash 1000m | 11 | 138.47 | 23.93 | 0.00 |
| altitude + orog mean + distance + orientation + ash 500m | 11 | 138.84 | 24.29 | 0.00 |
| altitude + orog mean + distance + orientation + ash 100m | 11 | 139.61 | 25.07 | 0.00 |
| altitude + orog min + distance + orientation + ash 100m | 11 | 141.15 | 26.60 | 0.00 |
| altitude + orog min + distance + orientation + ash 1000m | 11 | 141.34 | 26.79 | 0.00 |
| altitude + orog min + distance + orientation + ash 500m | 11 | 141.39 | 26.85 | 0.00 |

**Figure S1.** Non-parametric dispersion test and standard residuals plots for the final model obtained to assess changes in the total number of choughs recorded after the eruption. No significant problems were detected.


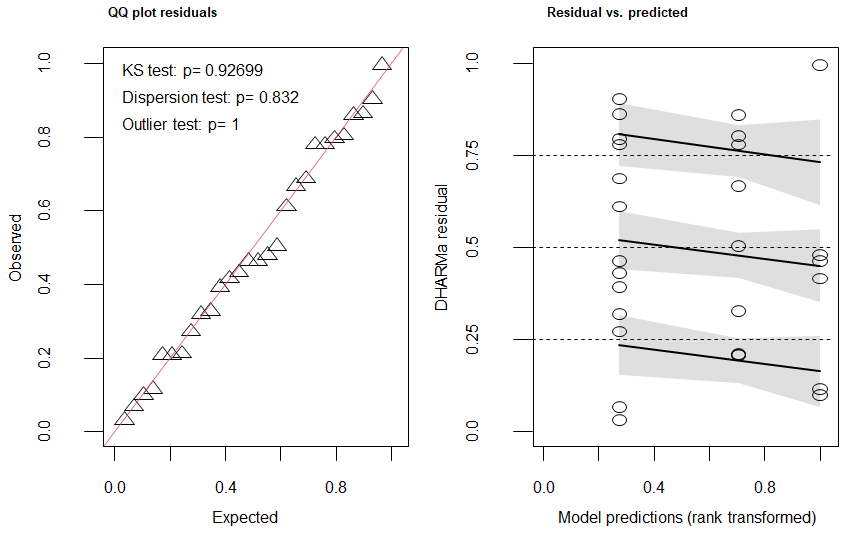


**Figure S2.** Non-parametric dispersion test and standard residuals plots for the final model obtained to assess factors affecting the number of total choughs after the eruption. No significant problems were detected.


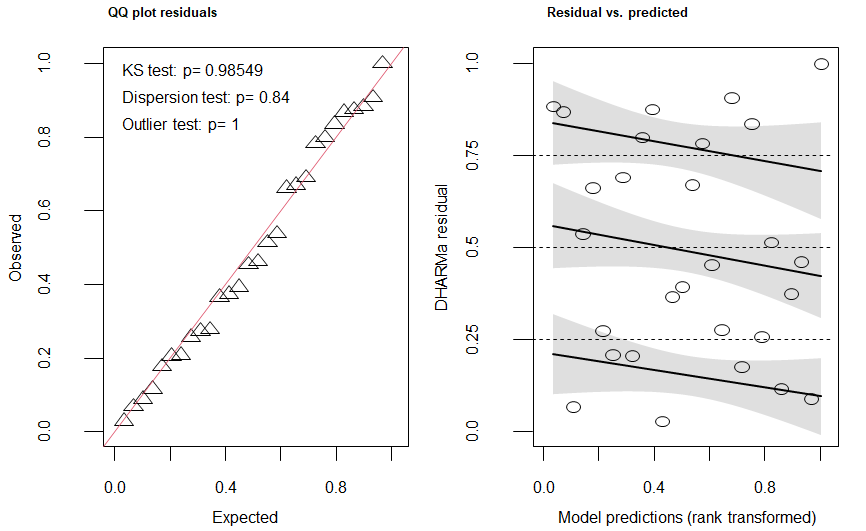


**Table 2.** Models obtained to assess the effect of the area where the breeding pairs were located (northern coast, central belt or southern quadrant), altitude, orientation (north, south, east, west, northeast, northeast, northeast, northwest, southeast, southwest), orography (measured as maximum, mean and minimum altitude with respect to the volcano: orog max, orog mean and orog min, respectively; see Methods), distance from the volcano (distance) and amount of ash accumulated in the surroundings (100m, 500m and 1,000m radii, ash 100m, ash 500m and ash 1000m, respectively) on the change in the total number of breeding pairs and the number of breeding pairs recorded after the eruption. df: number of estimated parameters, AICc: Akaike information criterion corrected for small sample sizes, ΔAICc: difference between each model and the best model (i.e. the model with the lowest AICc), weight: Akaike's weight.

| **Change in the number of breeding pairs** | | | | |  |
| --- | --- | --- | --- | --- | --- |
| **Model** | **df** | **AICc** | **∆AICc** | **w** | |
| Null | 2 | 63.93 | 0.00 | 0.23 | |
| orog min | 3 | 64.95 | 1.02 | 0.14 | |
| distance | 3 | 65.65 | 1.72 | 0.10 | |
| Altitude | 3 | 65.79 | 1.86 | 0.09 | |
| orog mean | 3 | 67.09 | 3.16 | 0.05 | |
| ash 1000m | 3 | 67.42 | 3.49 | 0.04 | |
| ash 100m | 3 | 67.42 | 3.49 | 0.04 | |
| ash 500m | 3 | 67.42 | 3.49 | 0.04 | |
| orog max | 3 | 67.79 | 3.87 | 0.03 | |
| orog mean + distance | 4 | 68.09 | 4.16 | 0.03 | |
| Area | 4 | 69.07 | 5.14 | 0.02 | |
| altitude + orog mean | 4 | 69.37 | 5.45 | 0.02 | |
| orog min + ash 1000m | 4 | 69.95 | 6.02 | 0.01 | |
| orog min + ash 500m | 4 | 69.97 | 6.04 | 0.01 | |
| orog min + ash 100m | 4 | 69.97 | 6.05 | 0.01 | |
| orog min + distance | 4 | 70.11 | 6.18 | 0.01 | |
| altitude + orog min | 4 | 70.11 | 6.18 | 0.01 | |
| orog max + distance | 4 | 70.11 | 6.18 | 0.01 | |
| distance + ash 1000m | 4 | 70.38 | 6.45 | 0.01 | |
| distance + ash 500m | 4 | 70.39 | 6.47 | 0.01 | |
| distance + ash 100m | 4 | 70.42 | 6.49 | 0.01 | |
| altitude + distance | 4 | 70.78 | 6.85 | 0.01 | |
| altitude + orog max | 4 | 70.86 | 6.93 | 0.01 | |
| altitude + ash 1000m | 4 | 71.03 | 7.10 | 0.01 | |
| altitude + ash 500m | 4 | 71.03 | 7.10 | 0.01 | |
| altitude + ash 100m | 4 | 71.03 | 7.10 | 0.01 | |
| orog mean + ash 500m | 4 | 71.72 | 7.79 | 0.00 | |
| orog mean + ash 100m | 4 | 71.72 | 7.79 | 0.00 | |
| orog mean + ash 1000m | 4 | 71.73 | 7.80 | 0.00 | |
| orog max + ash 1000m | 4 | 72.66 | 8.73 | 0.00 | |
| orog max + ash 100m | 4 | 72.66 | 8.73 | 0.00 | |
| orog max + ash 500m | 4 | 72.66 | 8.73 | 0.00 | |
| orog mean + distance + ash 1000m | 5 | 73.51 | 9.58 | 0.00 | |
| orog mean + distance + ash 500m | 5 | 73.67 | 9.74 | 0.00 | |
| orog mean + distance + ash 100m | 5 | 73.71 | 9.78 | 0.00 | |
| area + ash 1000m | 5 | 75.11 | 11.18 | 0.00 | |
| area + ash 100m | 5 | 75.16 | 11.23 | 0.00 | |
| area + ash 500m | 5 | 75.16 | 11.23 | 0.00 | |
| altitude + orog mean + distance | 5 | 75.42 | 11.49 | 0.00 | |
| area + orog mean | 5 | 75.71 | 11.78 | 0.00 | |
| area + orog min | 5 | 76.16 | 12.23 | 0.00 | |
| area + altitude | 5 | 76.20 | 12.27 | 0.00 | |
| area + distance | 5 | 76.37 | 12.44 | 0.00 | |
| area + orog max | 5 | 76.40 | 12.47 | 0.00 | |
| orog max + distance + ash 1000m | 5 | 76.59 | 12.66 | 0.00 | |
| orog max + distance + ash 500m | 5 | 76.65 | 12.72 | 0.00 | |
| orog max + distance + ash 100m | 5 | 76.69 | 12.76 | 0.00 | |
| altitude + orog mean + ash 1000m | 5 | 76.70 | 12.77 | 0.00 | |
| altitude + orog mean + ash 500m | 5 | 76.71 | 12.78 | 0.00 | |
| altitude + orog mean + ash 100m | 5 | 76.71 | 12.78 | 0.00 | |
| altitude + orog min + ash 1000m | 5 | 77.14 | 13.21 | 0.00 | |
| altitude + orog min + ash 500m | 5 | 77.15 | 13.22 | 0.00 | |
| altitude + orog min + ash 100m | 5 | 77.15 | 13.22 | 0.00 | |
| orog min + distance + ash 1000m | 5 | 77.28 | 13.35 | 0.00 | |
| orog min + distance + ash 500m | 5 | 77.30 | 13.37 | 0.00 | |
| orog min + distance + ash 100m | 5 | 77.31 | 13.38 | 0.00 | |
| altitude + orog min + distance | 5 | 77.36 | 13.43 | 0.00 | |
| altitude + orog max + distance | 5 | 77.43 | 13.50 | 0.00 | |
| altitude + distance + ash 1000m | 5 | 77.71 | 13.78 | 0.00 | |
| altitude + distance + ash 500m | 5 | 77.73 | 13.80 | 0.00 | |
| altitude + distance + ash 100m | 5 | 77.75 | 13.82 | 0.00 | |
| altitude + orog max + ash 100m | 5 | 78.19 | 14.26 | 0.00 | |
| altitude + orog max + ash 500m | 5 | 78.19 | 14.26 | 0.00 | |
| altitude + orog max + ash 1000m | 5 | 78.19 | 14.26 | 0.00 | |
| altitude + orog mean + distance + ash 1000m | 6 | 83.73 | 19.80 | 0.00 | |
| altitude + orog mean + distance + ash 500m | 6 | 83.83 | 19.90 | 0.00 | |
| altitude + orog mean + distance + ash 100m | 6 | 83.86 | 19.94 | 0.00 | |
| area + distance + ash 1000m | 6 | 84.83 | 20.90 | 0.00 | |
| area + distance + ash 500m | 6 | 84.97 | 21.05 | 0.00 | |
| area + distance + ash 100m | 6 | 85.03 | 21.10 | 0.00 | |
| area + orog mean + ash 1000m | 6 | 85.81 | 21.88 | 0.00 | |
| area + orog mean + ash 100m | 6 | 85.88 | 21.95 | 0.00 | |
| area + orog mean + ash 500m | 6 | 85.88 | 21.95 | 0.00 | |
| orientation | 6 | 86.02 | 22.09 | 0.00 | |
| area + altitude + ash 1000m | 6 | 86.03 | 22.10 | 0.00 | |
| area + orog max + ash 1000m | 6 | 86.07 | 22.15 | 0.00 | |
| area + altitude + ash 100m | 6 | 86.10 | 22.18 | 0.00 | |
| area + altitude + ash 500m | 6 | 86.11 | 22.18 | 0.00 | |
| area + orog min + ash 1000m | 6 | 86.11 | 22.18 | 0.00 | |
| area + orog max + ash 100m | 6 | 86.11 | 22.18 | 0.00 | |
| area + orog max + ash 500m | 6 | 86.12 | 22.19 | 0.00 | |
| area + orog min + ash 100m | 6 | 86.16 | 22.23 | 0.00 | |
| area + orog min + ash 500m | 6 | 86.16 | 22.23 | 0.00 | |
| area + orog mean + distance | 6 | 86.38 | 22.45 | 0.00 | |
| area + altitude + orog mean | 6 | 86.68 | 22.75 | 0.00 | |
| area + orog min + distance | 6 | 87.13 | 23.20 | 0.00 | |
| area + altitude + orog min | 6 | 87.15 | 23.23 | 0.00 | |
| area + altitude + distance | 6 | 87.20 | 23.27 | 0.00 | |
| area + altitude + orog max | 6 | 87.20 | 23.27 | 0.00 | |
| area + orog max + distance | 6 | 87.36 | 23.43 | 0.00 | |
| altitude + orog max + distance + ash 1000m | 6 | 87.46 | 23.53 | 0.00 | |
| altitude + orog max + distance + ash 500m | 6 | 87.51 | 23.58 | 0.00 | |
| altitude + orog max + distance + ash 100m | 6 | 87.56 | 23.63 | 0.00 | |
| altitude + orog min + distance + ash 1000m | 6 | 88.13 | 24.20 | 0.00 | |
| altitude + orog min + distance + ash 500m | 6 | 88.14 | 24.21 | 0.00 | |
| altitude + orog min + distance + ash 100m | 6 | 88.14 | 24.21 | 0.00 | |
| area + orog mean + distance + ash 1000m | 7 | 102.18 | 38.25 | 0.00 | |
| area + orog mean + distance + ash 500m | 7 | 102.42 | 38.49 | 0.00 | |
| area + orog mean + distance + ash 100m | 7 | 102.51 | 38.58 | 0.00 | |
| area + orog min + distance + ash 1000m | 7 | 103.02 | 39.09 | 0.00 | |
| area + altitude + distance + ash 1000m | 7 | 103.04 | 39.11 | 0.00 | |
| area + altitude + distance + ash 500m | 7 | 103.13 | 39.20 | 0.00 | |
| area + orog min + distance + ash 500m | 7 | 103.15 | 39.22 | 0.00 | |
| area + orog max + distance + ash 1000m | 7 | 103.16 | 39.23 | 0.00 | |
| area + altitude + distance + ash 100m | 7 | 103.19 | 39.26 | 0.00 | |
| area + orog min + distance + ash 100m | 7 | 103.22 | 39.29 | 0.00 | |
| area + orog max + distance + ash 500m | 7 | 103.31 | 39.38 | 0.00 | |
| area + orog max + distance + ash 100m | 7 | 103.36 | 39.43 | 0.00 | |
| altitude + orientation | 7 | 103.38 | 39.45 | 0.00 | |
| distance + orientation | 7 | 103.84 | 39.91 | 0.00 | |
| orog min + orientation | 7 | 103.88 | 39.95 | 0.00 | |
| area + altitude + orog mean + ash 1000m | 7 | 104.13 | 40.20 | 0.00 | |
| orog max + orientation | 7 | 104.20 | 40.27 | 0.00 | |
| area + altitude + orog mean + ash 100m | 7 | 104.20 | 40.28 | 0.00 | |
| area + altitude + orog mean + ash 500m | 7 | 104.21 | 40.28 | 0.00 | |
| area + altitude + orog min + ash 1000m | 7 | 104.23 | 40.30 | 0.00 | |
| orientation + ash 1000m | 7 | 104.28 | 40.35 | 0.00 | |
| orientation + ash 500m | 7 | 104.29 | 40.37 | 0.00 | |
| orientation + ash 100m | 7 | 104.30 | 40.37 | 0.00 | |
| area + altitude + orog max + ash 1000m | 7 | 104.31 | 40.38 | 0.00 | |
| area + altitude + orog min + ash 500m | 7 | 104.33 | 40.40 | 0.00 | |
| area + altitude + orog min + ash 100m | 7 | 104.34 | 40.41 | 0.00 | |
| orog mean + orientation | 7 | 104.35 | 40.42 | 0.00 | |
| area + altitude + orog max + ash 100m | 7 | 104.37 | 40.45 | 0.00 | |
| area + altitude + orog max + ash 500m | 7 | 104.38 | 40.45 | 0.00 | |
| area + altitude + orog mean + distance | 7 | 104.69 | 40.77 | 0.00 | |
| area + altitude + orog min + distance | 7 | 105.46 | 41.53 | 0.00 | |
| area + altitude + orog max + distance | 7 | 105.54 | 41.61 | 0.00 | |
| orog mean + distance + orientation | 8 | 137.30 | 73.37 | 0.00 | |
| area + altitude + orog mean + distance + ash 1000m | 8 | 137.43 | 73.50 | 0.00 | |
| area + altitude + orog mean + distance + ash 500m | 8 | 137.56 | 73.63 | 0.00 | |
| area + altitude + orog mean + distance + ash 100m | 8 | 137.72 | 73.79 | 0.00 | |
| altitude + orog mean + orientation | 8 | 139.48 | 75.55 | 0.00 | |
| altitude + orog min + orientation | 8 | 139.54 | 75.61 | 0.00 | |
| area + altitude + orog max + distance + ash 1000m | 8 | 139.66 | 75.73 | 0.00 | |
| area + altitude + orog min + distance + ash 1000m | 8 | 139.68 | 75.75 | 0.00 | |
| area + altitude + orog max + distance + ash 500m | 8 | 139.77 | 75.84 | 0.00 | |
| area + altitude + orog min + distance + ash 500m | 8 | 139.79 | 75.86 | 0.00 | |
| area + altitude + orog max + distance + ash 100m | 8 | 139.84 | 75.91 | 0.00 | |
| area + altitude + orog min + distance + ash 100m | 8 | 139.85 | 75.92 | 0.00 | |
| altitude + orog max + orientation | 8 | 140.01 | 76.08 | 0.00 | |
| altitude + distance + orientation | 8 | 140.03 | 76.10 | 0.00 | |
| altitude + orientation + ash 1000m | 8 | 140.04 | 76.11 | 0.00 | |
| altitude + orientation + ash 500m | 8 | 140.04 | 76.11 | 0.00 | |
| altitude + orientation + ash 100m | 8 | 140.04 | 76.11 | 0.00 | |
| orog max + distance + orientation | 8 | 140.16 | 76.23 | 0.00 | |
| distance + orientation + ash 1000m | 8 | 140.20 | 76.27 | 0.00 | |
| distance + orientation + ash 500m | 8 | 140.22 | 76.29 | 0.00 | |
| distance + orientation + ash 100m | 8 | 140.23 | 76.30 | 0.00 | |
| orog mean + orientation + ash 1000m | 8 | 140.50 | 76.57 | 0.00 | |
| orog min + distance + orientation | 8 | 140.51 | 76.58 | 0.00 | |
| orog min + orientation + ash 1000m | 8 | 140.51 | 76.58 | 0.00 | |
| orog min + orientation + ash 500m | 8 | 140.51 | 76.58 | 0.00 | |
| orog min + orientation + ash 100m | 8 | 140.51 | 76.58 | 0.00 | |
| orog mean + orientation + ash 500m | 8 | 140.61 | 76.68 | 0.00 | |
| orog mean + orientation + ash 100m | 8 | 140.65 | 76.72 | 0.00 | |
| orog max + orientation + ash 100m | 8 | 140.78 | 76.86 | 0.00 | |
| orog max + orientation + ash 500m | 8 | 140.79 | 76.86 | 0.00 | |
| orog max + orientation + ash 1000m | 8 | 140.81 | 76.88 | 0.00 | |
| altitude + orog min + orientation + ash 100m | 9 | 244.95 | 181.02 | 0.00 | |
| altitude + orog min + orientation + ash 500m | 9 | 244.99 | 181.06 | 0.00 | |
| altitude + orog min + orientation + ash 1000m | 9 | 245.03 | 181.10 | 0.00 | |
| altitude + orog mean + distance + orientation | 9 | 246.86 | 182.93 | 0.00 | |
| orog mean + distance + orientation + ash 100m | 9 | 246.89 | 182.96 | 0.00 | |
| orog mean + distance + orientation + ash 500m | 9 | 246.90 | 182.97 | 0.00 | |
| orog mean + distance + orientation + ash 1000m | 9 | 246.93 | 183.00 | 0.00 | |
| altitude + orog mean + orientation + ash 100m | 9 | 247.47 | 183.54 | 0.00 | |
| altitude + orog mean + orientation + ash 500m | 9 | 247.48 | 183.55 | 0.00 | |
| altitude + orog min + distance + orientation | 9 | 247.49 | 183.56 | 0.00 | |
| altitude + orog mean + orientation + ash 1000m | 9 | 247.50 | 183.57 | 0.00 | |
| altitude + orog max + orientation + ash 100m | 9 | 249.82 | 185.89 | 0.00 | |
| altitude + orog max + orientation + ash 500m | 9 | 249.83 | 185.91 | 0.00 | |
| altitude + orog max + orientation + ash 1000m | 9 | 249.87 | 185.94 | 0.00 | |
| orog min + distance + orientation + ash 1000m | 9 | 249.87 | 185.94 | 0.00 | |
| orog min + distance + orientation + ash 500m | 9 | 249.91 | 185.98 | 0.00 | |
| orog min + distance + orientation + ash 100m | 9 | 249.93 | 186.00 | 0.00 | |
| altitude + distance + orientation + ash 100m | 9 | 249.96 | 186.03 | 0.00 | |
| altitude + distance + orientation + ash 500m | 9 | 249.97 | 186.04 | 0.00 | |
| altitude + distance + orientation + ash 1000m | 9 | 249.98 | 186.05 | 0.00 | |
| altitude + orog max + distance + orientation | 9 | 249.98 | 186.05 | 0.00 | |
| orog max + distance + orientation + ash 1000m | 9 | 250.13 | 186.20 | 0.00 | |
| orog max + distance + orientation + ash 500m | 9 | 250.13 | 186.21 | 0.00 | |
| orog max + distance + orientation + ash 100m | 9 | 250.14 | 186.21 | 0.00 | |
| **Number of breeding pairs after the eruption** | | | | |  |
| **Model** | **df** | **AICc** | **∆AICc** | **w** | |
| area | 4 | 59.66 | 0.00 | 0.65 | |
| area + orog mean | 5 | 64.16 | 4.49 | 0.07 | |
| area + ash 1000m | 5 | 64.60 | 4.94 | 0.05 | |
| area + ash 100m | 5 | 64.70 | 5.04 | 0.05 | |
| area + ash 500m | 5 | 64.74 | 5.08 | 0.05 | |
| area + orog max | 5 | 66.08 | 6.42 | 0.03 | |
| area + orog min | 5 | 66.93 | 7.27 | 0.02 | |
| area + distance | 5 | 66.96 | 7.29 | 0.02 | |
| area + altitude | 5 | 66.99 | 7.33 | 0.02 | |
| area + orog mean + ash 100m | 6 | 67.63 | 7.96 | 0.01 | |
| area + orog mean + ash 1000m | 6 | 67.82 | 8.16 | 0.01 | |
| area + orog mean + ash 500m | 6 | 67.82 | 8.16 | 0.01 | |
| orog mean + distance | 4 | 71.86 | 12.19 | 0.00 | |
| orog min | 3 | 72.62 | 12.96 | 0.00 | |
| area + distance + ash 1000m | 6 | 73.07 | 13.41 | 0.00 | |
| area + orog max + ash 100m | 6 | 73.36 | 13.70 | 0.00 | |
| area + orog max + ash 1000m | 6 | 73.40 | 13.74 | 0.00 | |
| area + distance + ash 500m | 6 | 73.45 | 13.78 | 0.00 | |
| area + orog max + ash 500m | 6 | 73.48 | 13.82 | 0.00 | |
| area + distance + ash 100m | 6 | 73.51 | 13.84 | 0.00 | |
| distance | 3 | 74.53 | 14.87 | 0.00 | |
| area + altitude + orog mean | 6 | 74.56 | 14.90 | 0.00 | |
| area + orog mean + distance | 6 | 74.84 | 15.18 | 0.00 | |
| area + orog min + ash 1000m | 6 | 75.41 | 15.75 | 0.00 | |
| area + orog min + ash 100m | 6 | 75.48 | 15.82 | 0.00 | |
| area + orog min + ash 500m | 6 | 75.53 | 15.87 | 0.00 | |
| area + altitude + ash 1000m | 6 | 75.57 | 15.91 | 0.00 | |
| area + altitude + ash 100m | 6 | 75.64 | 15.97 | 0.00 | |
| area + altitude + ash 500m | 6 | 75.69 | 16.02 | 0.00 | |
| altitude + orog min | 4 | 75.90 | 16.23 | 0.00 | |
| null | 2 | 75.98 | 16.32 | 0.00 | |
| orientation | 6 | 76.25 | 16.58 | 0.00 | |
| altitude | 3 | 76.34 | 16.68 | 0.00 | |
| ash 1000m | 3 | 76.36 | 16.70 | 0.00 | |
| ash 100m | 3 | 76.40 | 16.74 | 0.00 | |
| ash 500m | 3 | 76.43 | 16.76 | 0.00 | |
| area + altitude + orog max | 6 | 76.99 | 17.33 | 0.00 | |
| area + orog max + distance | 6 | 77.08 | 17.41 | 0.00 | |
| orog max + distance | 4 | 77.13 | 17.47 | 0.00 | |
| orog min + ash 100m | 4 | 77.53 | 17.86 | 0.00 | |
| orog min + ash 500m | 4 | 77.54 | 17.88 | 0.00 | |
| orog min + ash 1000m | 4 | 77.55 | 17.89 | 0.00 | |
| orog min + distance | 4 | 77.68 | 18.02 | 0.00 | |
| area + altitude + orog min | 6 | 77.88 | 18.22 | 0.00 | |
| area + orog min + distance | 6 | 77.89 | 18.22 | 0.00 | |
| area + altitude + distance | 6 | 77.96 | 18.29 | 0.00 | |
| altitude + orog mean + distance | 5 | 78.32 | 18.66 | 0.00 | |
| altitude + orog mean | 4 | 78.50 | 18.84 | 0.00 | |
| orog mean | 3 | 78.75 | 19.09 | 0.00 | |
| orog mean + distance + ash 1000m | 5 | 79.01 | 19.34 | 0.00 | |
| orog mean + distance + ash 500m | 5 | 79.05 | 19.38 | 0.00 | |
| orog mean + distance + ash 100m | 5 | 79.07 | 19.40 | 0.00 | |
| orog mean + ash 1000m | 4 | 79.33 | 19.67 | 0.00 | |
| orog mean + ash 100m | 4 | 79.34 | 19.68 | 0.00 | |
| orog mean + ash 500m | 4 | 79.34 | 19.68 | 0.00 | |
| distance + ash 100m | 4 | 79.64 | 19.98 | 0.00 | |
| distance + ash 1000m | 4 | 79.65 | 19.98 | 0.00 | |
| distance + ash 500m | 4 | 79.66 | 19.99 | 0.00 | |
| altitude + distance | 4 | 79.76 | 20.10 | 0.00 | |
| orog max | 3 | 79.81 | 20.14 | 0.00 | |
| altitude + ash 100m | 4 | 80.11 | 20.44 | 0.00 | |
| altitude + ash 500m | 4 | 80.15 | 20.48 | 0.00 | |
| altitude + ash 1000m | 4 | 80.17 | 20.51 | 0.00 | |
| altitude + orog max | 4 | 81.26 | 21.60 | 0.00 | |
| orog max + ash 1000m | 4 | 81.43 | 21.76 | 0.00 | |
| orog max + ash 100m | 4 | 81.45 | 21.79 | 0.00 | |
| orog max + ash 500m | 4 | 81.48 | 21.82 | 0.00 | |
| altitude + orog min + distance | 5 | 83.00 | 23.34 | 0.00 | |
| altitude + orog min + ash 1000m | 5 | 83.10 | 23.44 | 0.00 | |
| altitude + orog min + ash 500m | 5 | 83.12 | 23.46 | 0.00 | |
| altitude + orog min + ash 100m | 5 | 83.13 | 23.46 | 0.00 | |
| orog min + distance + ash 100m | 5 | 83.68 | 24.01 | 0.00 | |
| orog min + distance + ash 500m | 5 | 83.68 | 24.02 | 0.00 | |
| orog min + distance + ash 1000m | 5 | 83.74 | 24.08 | 0.00 | |
| altitude + orog mean + ash 100m | 5 | 83.77 | 24.11 | 0.00 | |
| altitude + orog mean + ash 500m | 5 | 83.81 | 24.15 | 0.00 | |
| altitude + orog mean + ash 1000m | 5 | 83.93 | 24.27 | 0.00 | |
| altitude + orog max + distance | 5 | 84.18 | 24.51 | 0.00 | |
| orog max + distance + ash 100m | 5 | 84.44 | 24.78 | 0.00 | |
| orog max + distance + ash 500m | 5 | 84.45 | 24.79 | 0.00 | |
| orog max + distance + ash 1000m | 5 | 84.46 | 24.79 | 0.00 | |
| area + orog mean + distance + ash 1000m | 7 | 84.66 | 24.99 | 0.00 | |
| area + orog mean + distance + ash 100m | 7 | 84.73 | 25.06 | 0.00 | |
| area + orog mean + distance + ash 500m | 7 | 84.82 | 25.16 | 0.00 | |
| area + altitude + orog mean + ash 1000m | 7 | 85.42 | 25.75 | 0.00 | |
| area + altitude + orog mean + ash 100m | 7 | 85.46 | 25.79 | 0.00 | |
| area + altitude + orog mean + ash 500m | 7 | 85.61 | 25.94 | 0.00 | |
| altitude + orog max + ash 100m | 5 | 86.61 | 26.95 | 0.00 | |
| altitude + orog max + ash 500m | 5 | 86.67 | 27.01 | 0.00 | |
| altitude + orog max + ash 1000m | 5 | 86.75 | 27.09 | 0.00 | |
| altitude + distance + ash 100m | 5 | 86.97 | 27.30 | 0.00 | |
| altitude + distance + ash 1000m | 5 | 86.98 | 27.31 | 0.00 | |
| altitude + distance + ash 500m | 5 | 86.98 | 27.32 | 0.00 | |
| orog mean + orientation | 7 | 87.63 | 27.97 | 0.00 | |
| altitude + orog mean + distance + ash 500m | 6 | 88.14 | 28.48 | 0.00 | |
| altitude + orog mean + distance + ash 1000m | 6 | 88.14 | 28.48 | 0.00 | |
| altitude + orog mean + distance + ash 100m | 6 | 88.19 | 28.52 | 0.00 | |
| orog max + orientation | 7 | 89.54 | 29.88 | 0.00 | |
| area + orog max + distance + ash 1000m | 7 | 89.75 | 30.08 | 0.00 | |
| area + altitude + distance + ash 1000m | 7 | 89.75 | 30.08 | 0.00 | |
| area + altitude + distance + ash 500m | 7 | 89.86 | 30.20 | 0.00 | |
| area + altitude + distance + ash 100m | 7 | 89.92 | 30.26 | 0.00 | |
| area + orog max + distance + ash 100m | 7 | 89.97 | 30.31 | 0.00 | |
| area + orog max + distance + ash 500m | 7 | 90.01 | 30.35 | 0.00 | |
| area + orog min + distance + ash 1000m | 7 | 90.11 | 30.45 | 0.00 | |
| area + orog min + distance + ash 500m | 7 | 90.44 | 30.78 | 0.00 | |
| area + orog min + distance + ash 100m | 7 | 90.56 | 30.90 | 0.00 | |
| distance + orientation | 7 | 90.94 | 31.28 | 0.00 | |
| altitude + orientation | 7 | 91.34 | 31.68 | 0.00 | |
| orog min + orientation | 7 | 91.48 | 31.82 | 0.00 | |
| area + altitude + orog mean + distance | 7 | 91.48 | 31.82 | 0.00 | |
| area + altitude + orog max + ash 100m | 7 | 91.70 | 32.03 | 0.00 | |
| area + altitude + orog max + ash 1000m | 7 | 91.72 | 32.06 | 0.00 | |
| area + altitude + orog max + ash 500m | 7 | 91.82 | 32.15 | 0.00 | |
| altitude + orog min + distance + ash 1000m | 6 | 93.15 | 33.49 | 0.00 | |
| altitude + orog min + distance + ash 500m | 6 | 93.20 | 33.54 | 0.00 | |
| orientation + ash 1000m | 7 | 93.23 | 33.56 | 0.00 | |
| altitude + orog min + distance + ash 100m | 6 | 93.23 | 33.57 | 0.00 | |
| orientation + ash 500m | 7 | 93.34 | 33.68 | 0.00 | |
| orientation + ash 100m | 7 | 93.40 | 33.74 | 0.00 | |
| area + altitude + orog min + ash 1000m | 7 | 93.64 | 33.97 | 0.00 | |
| area + altitude + orog min + ash 100m | 7 | 93.76 | 34.10 | 0.00 | |
| area + altitude + orog min + ash 500m | 7 | 93.80 | 34.14 | 0.00 | |
| altitude + orog max + distance + ash 1000m | 6 | 95.14 | 35.47 | 0.00 | |
| altitude + orog max + distance + ash 500m | 6 | 95.14 | 35.48 | 0.00 | |
| altitude + orog max + distance + ash 100m | 6 | 95.15 | 35.49 | 0.00 | |
| area + altitude + orog max + distance | 7 | 95.28 | 35.61 | 0.00 | |
| area + altitude + orog min + distance | 7 | 96.06 | 36.40 | 0.00 | |
| orog mean + orientation + ash 1000m | 8 | 114.75 | 55.08 | 0.00 | |
| orog mean + orientation + ash 500m | 8 | 115.40 | 55.74 | 0.00 | |
| orog mean + orientation + ash 100m | 8 | 115.67 | 56.01 | 0.00 | |
| orog max + orientation + ash 1000m | 8 | 118.91 | 59.25 | 0.00 | |
| orog max + orientation + ash 500m | 8 | 119.17 | 59.51 | 0.00 | |
| orog max + orientation + ash 100m | 8 | 119.30 | 59.63 | 0.00 | |
| area + altitude + orog mean + distance + ash 1000m | 8 | 121.32 | 61.65 | 0.00 | |
| area + altitude + orog mean + distance + ash 100m | 8 | 121.39 | 61.73 | 0.00 | |
| area + altitude + orog mean + distance + ash 500m | 8 | 121.49 | 61.82 | 0.00 | |
| orog mean + distance + orientation | 8 | 123.50 | 63.84 | 0.00 | |
| altitude + orog mean + orientation | 8 | 123.72 | 64.06 | 0.00 | |
| altitude + orog max + orientation | 8 | 125.48 | 65.81 | 0.00 | |
| area + altitude + orog max + distance + ash 1000m | 8 | 125.52 | 65.86 | 0.00 | |
| area + altitude + orog max + distance + ash 100m | 8 | 125.55 | 65.88 | 0.00 | |
| area + altitude + orog max + distance + ash 500m | 8 | 125.59 | 65.92 | 0.00 | |
| orog max + distance + orientation | 8 | 126.19 | 66.52 | 0.00 | |
| area + altitude + orog min + distance + ash 1000m | 8 | 126.38 | 66.72 | 0.00 | |
| area + altitude + orog min + distance + ash 500m | 8 | 126.52 | 66.85 | 0.00 | |
| area + altitude + orog min + distance + ash 100m | 8 | 126.58 | 66.92 | 0.00 | |
| distance + orientation + ash 1000m | 8 | 127.21 | 67.54 | 0.00 | |
| distance + orientation + ash 500m | 8 | 127.23 | 67.56 | 0.00 | |
| distance + orientation + ash 100m | 8 | 127.23 | 67.57 | 0.00 | |
| altitude + distance + orientation | 8 | 127.31 | 67.65 | 0.00 | |
| altitude + orientation + ash 500m | 8 | 127.47 | 67.80 | 0.00 | |
| altitude + orientation + ash 100m | 8 | 127.47 | 67.80 | 0.00 | |
| altitude + orientation + ash 1000m | 8 | 127.47 | 67.81 | 0.00 | |
| orog min + distance + orientation | 8 | 127.61 | 67.94 | 0.00 | |
| altitude + orog min + orientation | 8 | 127.95 | 68.29 | 0.00 | |
| orog min + orientation + ash 1000m | 8 | 128.11 | 68.44 | 0.00 | |
| orog min + orientation + ash 500m | 8 | 128.11 | 68.45 | 0.00 | |
| orog min + orientation + ash 100m | 8 | 128.11 | 68.45 | 0.00 | |
| altitude + orog mean + distance + orientation | 9 | 218.65 | 158.98 | 0.00 | |
| orog mean + distance + orientation + ash 1000m | 9 | 223.88 | 164.22 | 0.00 | |
| orog mean + distance + orientation + ash 500m | 9 | 224.13 | 164.46 | 0.00 | |
| orog mean + distance + orientation + ash 100m | 9 | 224.20 | 164.54 | 0.00 | |
| altitude + orog mean + orientation + ash 1000m | 9 | 224.62 | 164.96 | 0.00 | |
| altitude + orog mean + orientation + ash 500m | 9 | 225.14 | 165.48 | 0.00 | |
| altitude + orog mean + orientation + ash 100m | 9 | 225.34 | 165.68 | 0.00 | |
| altitude + orog max + orientation + ash 100m | 9 | 227.76 | 168.10 | 0.00 | |
| altitude + orog max + orientation + ash 500m | 9 | 227.87 | 168.21 | 0.00 | |
| altitude + orog max + orientation + ash 1000m | 9 | 228.09 | 168.43 | 0.00 | |
| orog max + distance + orientation + ash 100m | 9 | 228.58 | 168.92 | 0.00 | |
| orog max + distance + orientation + ash 500m | 9 | 228.61 | 168.95 | 0.00 | |
| orog max + distance + orientation + ash 1000m | 9 | 228.65 | 168.98 | 0.00 | |
| altitude + orog max + distance + orientation | 9 | 232.93 | 173.27 | 0.00 | |
| altitude + orog min + orientation + ash 1000m | 9 | 235.23 | 175.57 | 0.00 | |
| altitude + orog min + orientation + ash 500m | 9 | 235.27 | 175.61 | 0.00 | |
| altitude + orog min + orientation + ash 100m | 9 | 235.32 | 175.65 | 0.00 | |
| altitude + orog min + distance + orientation | 9 | 235.36 | 175.70 | 0.00 | |
| orog min + distance + orientation + ash 1000m | 9 | 236.57 | 176.91 | 0.00 | |
| orog min + distance + orientation + ash 500m | 9 | 236.63 | 176.97 | 0.00 | |
| orog min + distance + orientation + ash 100m | 9 | 236.64 | 176.98 | 0.00 | |
| altitude + distance + orientation + ash 1000m | 9 | 237.20 | 177.53 | 0.00 | |
| altitude + distance + orientation + ash 500m | 9 | 237.22 | 177.56 | 0.00 | |
| altitude + distance + orientation + ash 100m | 9 | 237.23 | 177.56 | 0.00 | |

**Figure S3.** Non-parametric dispersion test and standard residuals plots for the final model obtained to assess changes in the number of breeding pairs after the eruption. No significant problems were detected.


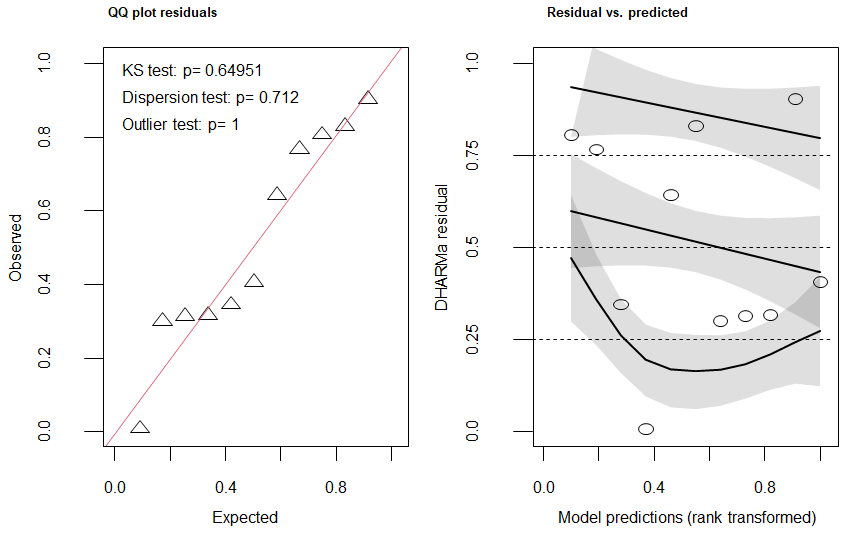


**Figure S4.** Non-parametric dispersion test and standard residuals plots for the final model obtained to assess factors affecting the number of breeding pairs after the eruption. No significant problems were detected.


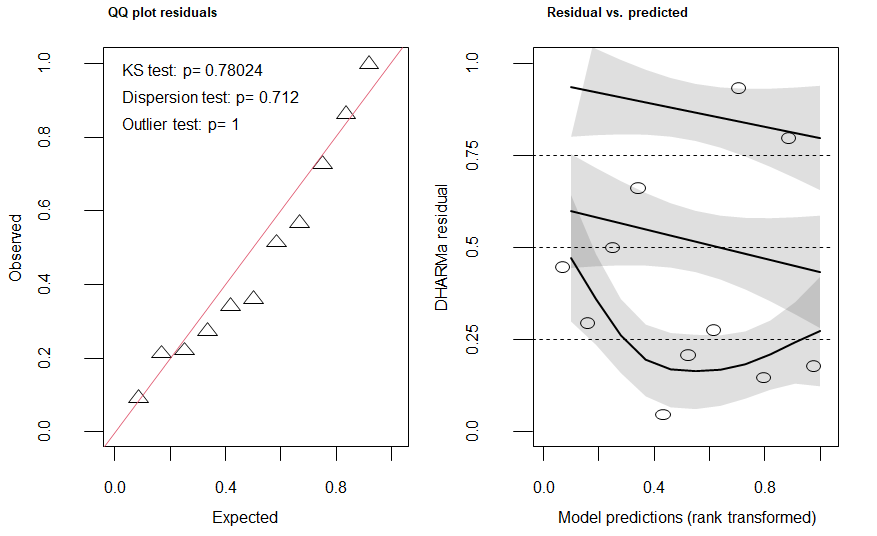

Supplement: Supplemental Information 2 [file peerj-12-18071-s002.docx]
